# Facially expressive humanoid robotic face

**DOI:** 10.1016/j.ohx.2020.e00117

**Published:** 2020-06-12

**Authors:** Zanwar Faraj, Mert Selamet, Carlos Morales, Patricio Torres, Maimuna Hossain, Boyuan Chen, Hod Lipson

**Affiliations:** Department of Mechanical Engineering, Columbia University, New York, NY, United States

**Keywords:** Humanoid, Face robot, Facial expressions, Emotions, Artificial intelligence

## Abstract

Realistic humanoid robots have emerged in the last two decades but the emotional intelligence of these machines has been limited. To teach humanoids how to emotionally communicate with humans, researchers have been increasingly relying on machine learning algorithms. While the software used to implement machine learning algorithms is largely open source, facially expressive humanoid robots are expensive and inaccessible to most people, thus limiting the number of researchers in this field. This paper aims to aid potential artificial intelligence researchers by providing a relatively inexpensive, open-source robot that can serve as a platform for research into emotional communication between humans and machines. Eva, the robot described in this paper, is an adult-sized humanoid head that can emulate human facial expressions, head movements, and speech through the use of 25 muscles, including 12 facial muscles that can produce a maximum skin displacement of 15 mm.

## Graphical abstract

**Figure:**
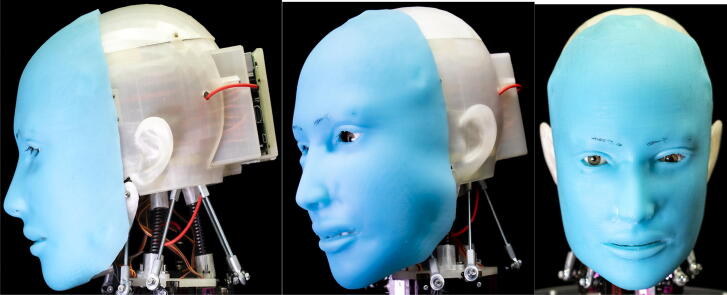

Specifications table

**Table:** 

Hardware name	Eva
Subject area	Engineering and Material Science
Hardware type	• Mechanical engineering and materials science • Electrical engineering and computer science
Open Source License	BSD 3-Clause
Cost of Hardware	$900
Source File Repository	https://doi.org/10.5281/zenodo.3539723

## 1 Hardware in context

The advancement of robotics technology in the last two decades has resulted in the development of numerous humanoid robots. Some of these humanoids are almost indistinguishable from humans in terms of their physical appearance and facial movements. Because of their ability to communicate emotions through their facial expressions, these realistic humanoids are often created by organizations to conduct emotional artificial intelligence research.

One such organization is Hanson Robotics, which is home to some of the world’s most lifelike humanoid robots [1]. Since its creation of the Albert Einstein HUBO in 2005, the first walking robot with realistic, humanlike expressions, Hanson Robotics has been a leader in the field of humanoid robotics and artificial intelligence. Sophia, the company’s most advanced robot, is capable of emulating 62 facial expressions and utilizes machine learning algorithms to learn how to communicate properly with humans.

Another notable organization in the field of humanoid robotics is Hiroshi Ishiguro Laboratories [2]. This Japanese research institute specializes in creating “Geminoids”, realistic humanoid robots that resemble a specific person rather than a generic human face. The institute is currently conducting several research projects, including a novel project on the “teleoperation”, or remote operation, of Geminoids. In this project, a facial recognition system is used to allow a Geminoid to mimic the facial expressions and mouth movements of a remote human operator, ideally the human on which the Geminoid was modeled after. In this manner, the Geminoid can mimic natural human behavior more closely than if it was solely operated by artificial intelligence software.

Despite the success of these organizations, their impact is limited by the fact that their hardware is proprietary, rather than open source. This prevents researchers who are unaffiliated with these organizations from using these humanoids to conduct their own research. Given the lack of open-source alternatives, unaffiliated researchers are generally forced to create their own humanoid robots to perform independent research. As building a realistic humanoid from scratch requires a considerable amount of time and resources, advancement in the field of emotional artificial intelligence has been hindered by this high barrier to entry.

**Fig. 1:**
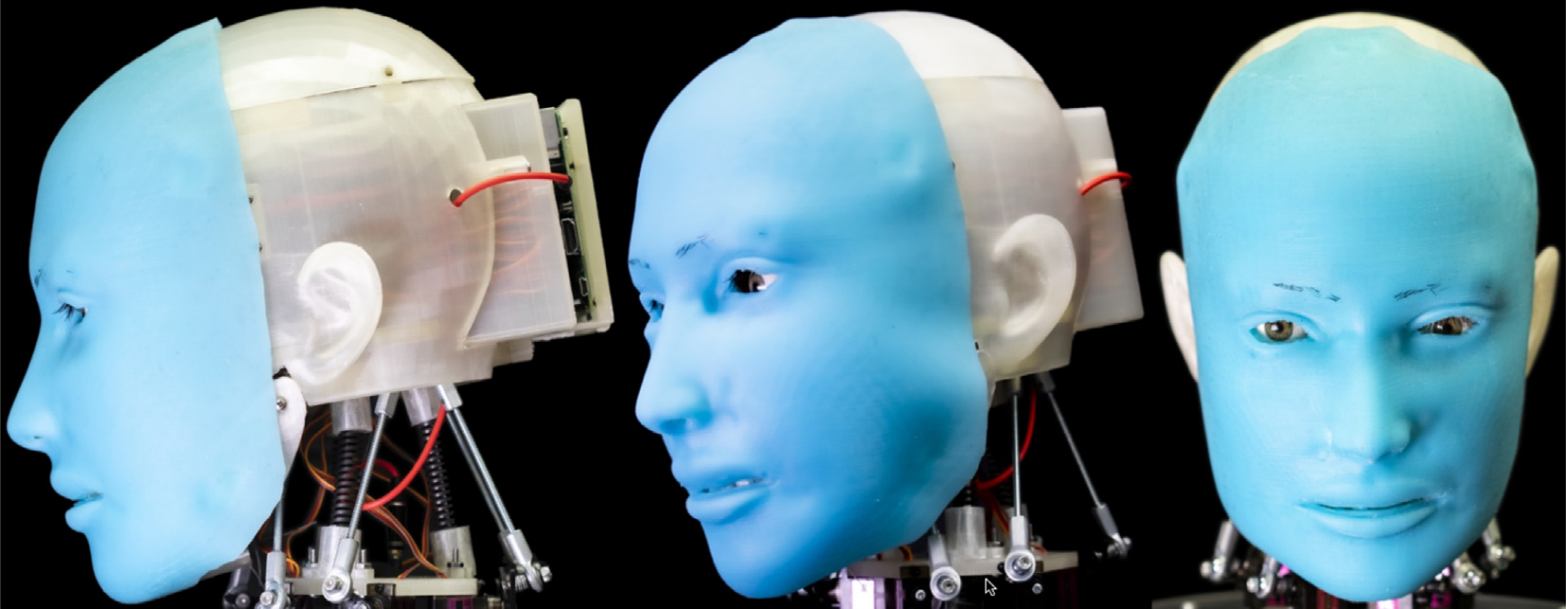
Three views of current Eva prototype (photo credit: Jonathan Blutinger).

Eva, the humanoid presented in this paper, was designed to tackle this problem by serving as an accessible, open-source robot that artificial intelligence researchers can use to conduct their own research into human-robot communication. Three views of the current Eva prototype are provided in Fig. 1. Eva can express the emotions of joy, sadness, surprise, anger, fear, and disgust through six unique facial expressions actuated by servo motors. Eva can also move its eyes, open and close its eyelids, nod and turn its head, and emulate speech by coordinating the opening and closing of its mouth with text-to-speech audio emitted through an onboard speaker. Eva is controlled by running Python files on the onboard Raspberry Pi. As Python is one of the leading programming languages for machine learning, researchers can readily make use of pre-existing machining learning libraries such as scikit-learn.

There are numerous facially expressive humanoid robots in existence, but Eva appears to be the only one that is open source. While Eva is not as sophisticated as the robots developed by organizations such as Hanson Robotics and Hiroshi Ishiguro Laboratories, Eva is still capable of realistically emulating human facial expressions and head movements. A thorough comparison of Eva to similar facially expressive humanoids robots is provided in Table 1. For an open-source, human-size, facially expressive humanoid robot, there appears to be no current alterative to Eva.

**Table 1:** Comparison of Eva to other facially expressive humanoid robots.

Humanoid	Image	Number of actuators	Actuator type(s)	Mask material	Weight (kg)	Head Size	With body (Y/N)	Open source (Y/N)	Consumer product (Y/N)	Cost ($)
Eva (this paper)		25	Servo motor	Silicone	3	Adult	N	**Yes**	N	900
Face Robot [3]		24	FMA, stepper motor, air cylinder	Silicone	N/A	Adult	N	N	N	N/A
Face Robot [4]		N/A	McKibben actuator, DC motor	Urethane resin	1.5	Adult	N	N	N	N/A
Albert HUBO [5]		66 (31 in head, 35 in body)	Servo motor	Frubber	57	Adult	Y	N	N	N/A
Face Robot [6]		39	McKibben actuator	Urethane resin	N/A	Adult	N	N	N	N/A
Face Robot [7]		7	Servo motor	Urethane resin	N/A	Child	N	N	N	N/A
HRP-4C [8]		44	Servo motor	Silicone	46	Adult	Y	N	N	N/A
Geminoid DK [9]		N/A	Pneumatic	Silicone	40	Adult	Y	N	N	165,000
Erica [10]		44	Pneumatic	Silicone	N/A	Adult	Y	N	N	N/A
Geminoid HI-2 [11]		50	Pneumatic	Silicone	N/A	Adult	Y	N	N	N/A
Sophia [1]		34	Servo motor	Frubber	20	Adult	Y	N	N	N/A
Professor Einstein [1]		9	Servo motor	Frubber	N/A	Miniature	Y	N	Y	200
Little Sophia [1]		N/A	Servo motor	Frubber	N/A	Miniature	Y	N	Y	149
Telenoid R4 [12]		9	Servo motor	Polyvinyl chloride	3.5	Child	Y	N	Y	8,000

As shown in Table 1, human-size facially expressive robots are largely inaccessible to consumers but there are a few miniature humanoid robots available for purchase. In particular, Hanson Robotics’ Professor Einstein is a commercially available humanoid toy that is capable of walking, talking, and making facial expressions. Professor Einstein’s facial expressions are surprisingly realistic but it is only capable of making a few distinct expressions as its face is only actuated by five motors. Its functionality is also largely confined to a proprietary app by Hanson Robotics, which greatly limits its usefulness to researchers. Finally, Professor Einstein’s miniature size prevents it from eliciting the strong visceral response from humans that a human-size robot is capable of. Hanson Robotics’ latest miniature robot, Little Sophia, could hold more value for researchers. It is a facially expressive humanoid similar to Professor Einstein that is set to release in late 2020. While Little Sophia can be programmed in Python and appears to be capable of making a wider range of facial expressions than its predecessor, its utility is still hindered by its miniature size and the need to access its functionality through a proprietary companion app from Hanson Robotics.

Another commercially available humanoid is Telenoid R4 developed by Hiroshi Ishiguro Laboratories. Unlike Professor Einstein or Little Sophia, Telenoid R4 does not attempt to closely resemble a human, lacking ears, hands, legs, and hair. Telenoid R4 is also much larger and more expensive than Professor Einstein or Little Sophia. While Telenoid R4 is well suited for teleoperation applications, such as providing a physical presence for individuals when remotely calling a family member, its limited facial expressions restrict its usefulness for researchers.

## 2 Hardware description

### 2.1 Overview of hardware components

Eva is comprised of four main mechanisms: the mask actuation mechanism, the jaw, the eyes, and the neck. Important auxiliary components include the skull, the base platform, the speaker, the cooling fan, the Raspberry Pi and servo hats, the power supply, and the external monitor, keyboard, and mouse. Eva has 25 muscles in the form of servo motors, including 12 facial muscles that can produce a maximum skin displacement of 15 mm.

**Fig. 2:**
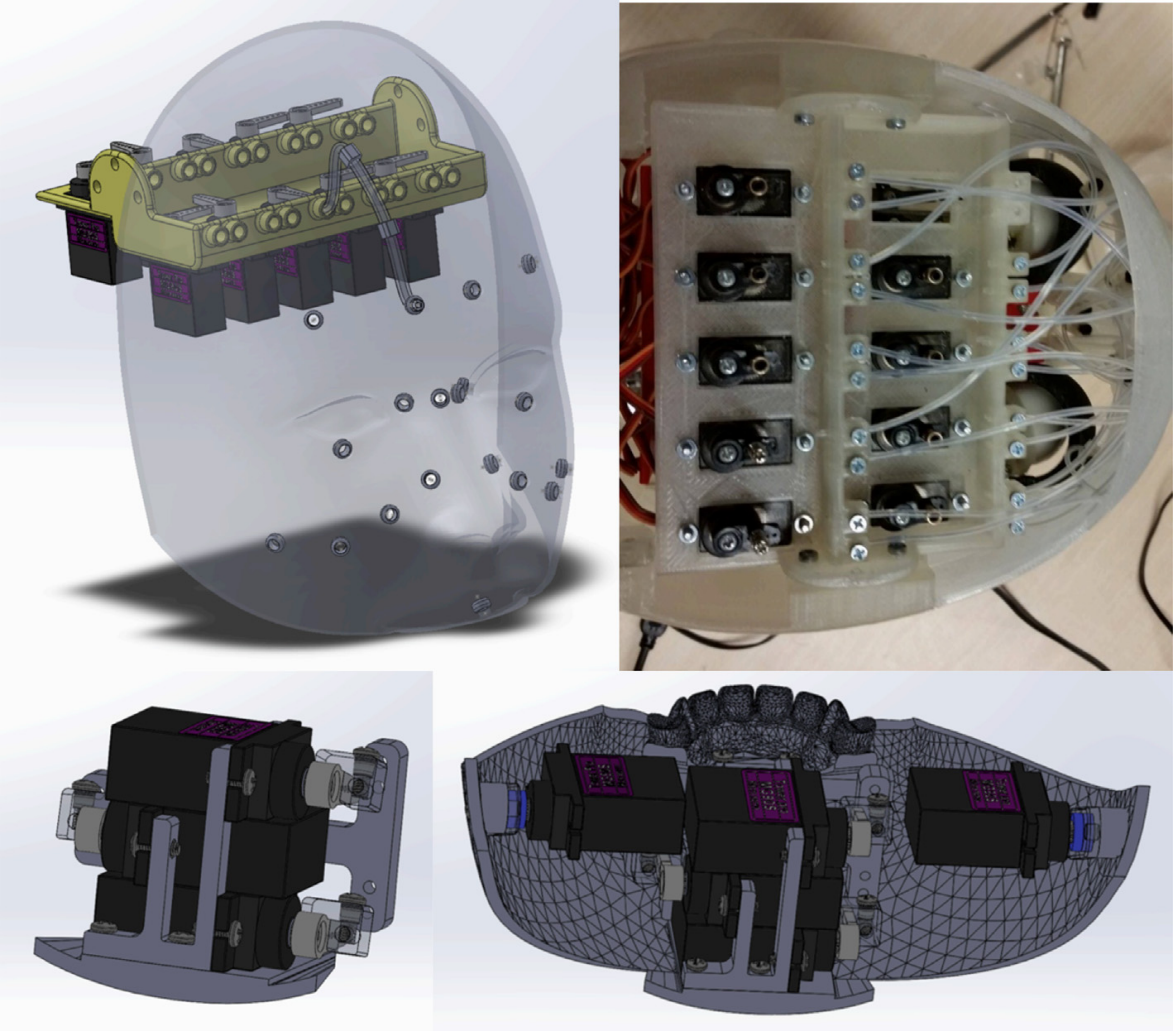
(top left) Bowden tube mechanism that provides a path from the skull to the servos, (top right) top servo bank located at the top of the skull, (bottom left) bottom servo bank shown in isolation, (bottom right) bottom servo bank attached to the jaw.

The mask actuation mechanism makes use of 12 MG90S servo motors, two 3D printed servo banks for housing the servo motors, a custom silicone mask, a 3D printed skull for supporting the mask, and steel wires fed through Teflon Bowden tubes. Fig. 2 illustrates how the Teflon Bowden tubes provide a path between the outer surface of the skull and the servo banks located inside the skull. Steel wires are fed through the Bowden tubes, with one end of each wire attached to the servo horn of the corresponding servo motor and the other end attached to a point on the inner surface of the mask. Each servo motor is connected to two steel wires which are attached to symmetric points on the left and right sides of the mask. This symmetry ensures that both sides of the mask are actuated when Eva makes a facial expression. Fig. 2 also depicts the top servo bank (which houses nine servos and is located at the top of the skull) and the bottom servo bank (which houses three servos and is located on the jaw). The top servo bank is responsible for actuating the wires connected to the upper portion of the mask while the bottom servo bank is responsible for actuating the wires connected to the lower portion of the mask near the jaw.

**Fig. 3:**
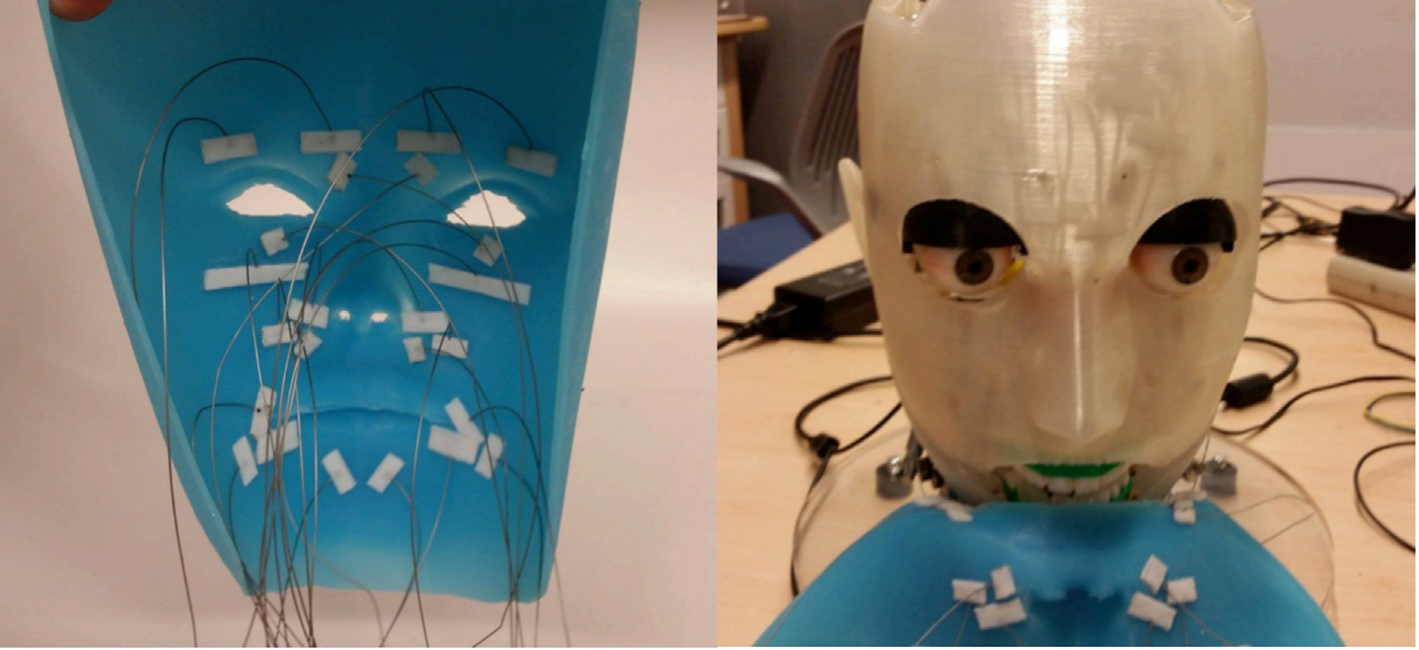
(left) Steel wires attached to silicone mask with cloth and adhesive, (right) silicone mask partially wrapped around skull with steel wires attached to servos in bottom servo bank.

Fig. 3 shows how the steel wires are attached to the inner surface of the silicone mask with strips of cloth and adhesive. These wires are fed through the tubes and attached to the various servo motors inside the skull. The silicone mask is made from Smooth-On EcoFlex 00-30, a substance that closely simulates the material properties of human skin. To generate a facial expression, a specific subset of the 12 servo motors are actuated, which causes the steel wires to be pulled and the mask to be deformed in a way that simulates how facial muscles deform the skin when making expressions. The skull shown in Fig. 3 supports the mask so that pulling on the wires does not cause the mask to collapse inwards.

**Fig. 4:**
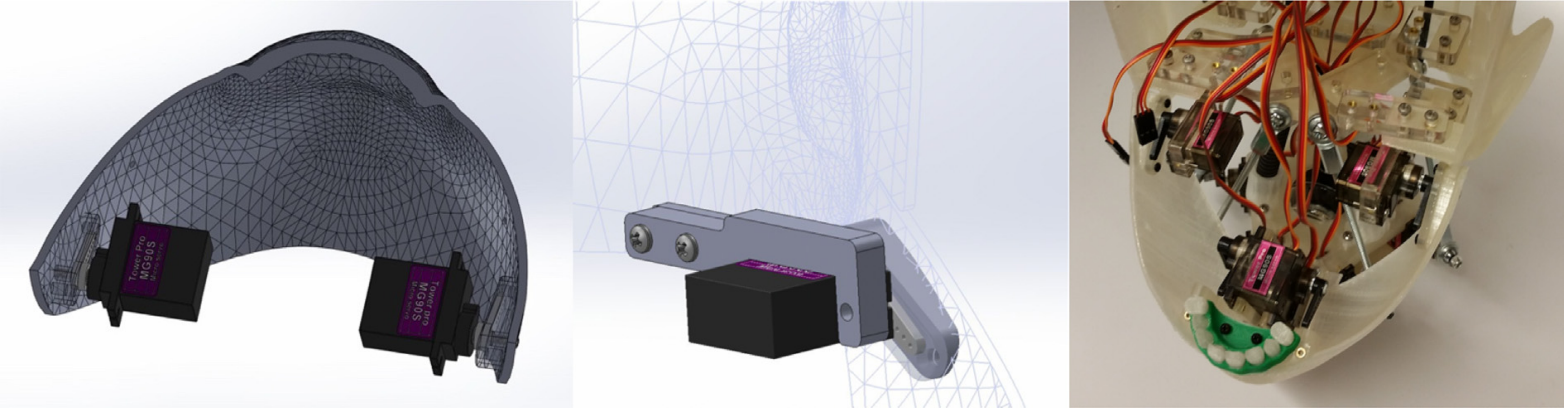
(left) 3D printed jaw and two jaw servos, (center) interface of the jaw to the skull, (right) final jaw mechanism in the full assembly.

The jaw mechanism makes use of two MG90S servos and a 3D printed jaw, as shown in Fig. 4. The servos themselves are attached to the stationary skull with screws while the jaw is screwed securely to the two servo horns. Thus, when the servos are actuated, the servos remain stationary while the jaw rotates about the axis through the servo horns.

**Fig. 5:**
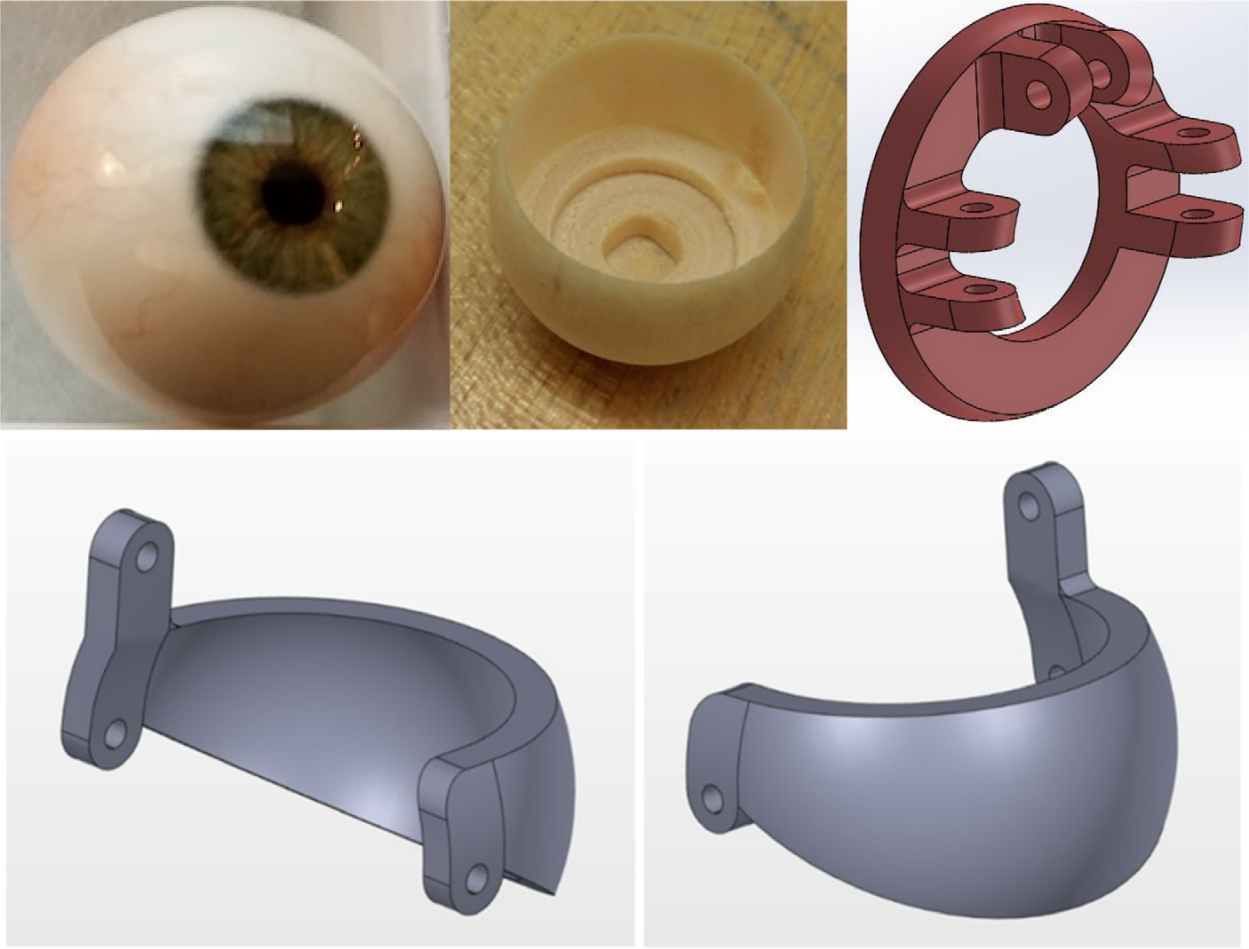
(top left) Eyeball replica, (top center) machined eyeball, (top right) eyeball attachment for M2 ball joints, (bottom) 3D printed eyelids.

The eyeballs and eyelids are comprised of five MG90S servo motors, two machined eyeball replicas, two 3D printed eyeball attachments, two 3D printed eyelids, an acrylic plate, M2 ball joints, threaded connecting rods, and several 3D printed attachments. Fig. 5 shows an un-machined eyeball replica, an eyeball after machining out the core, and a 3D printed eyeball attachment snapped onto the machined eyeball to provide an interface for the M2 ball joints and threaded connecting rods. Fig. 5 also depicts the 3D printed eyelids that partially envelope the eyeballs. A unique eyelid design is used for both the left and right eyelids.

**Fig. 6:**
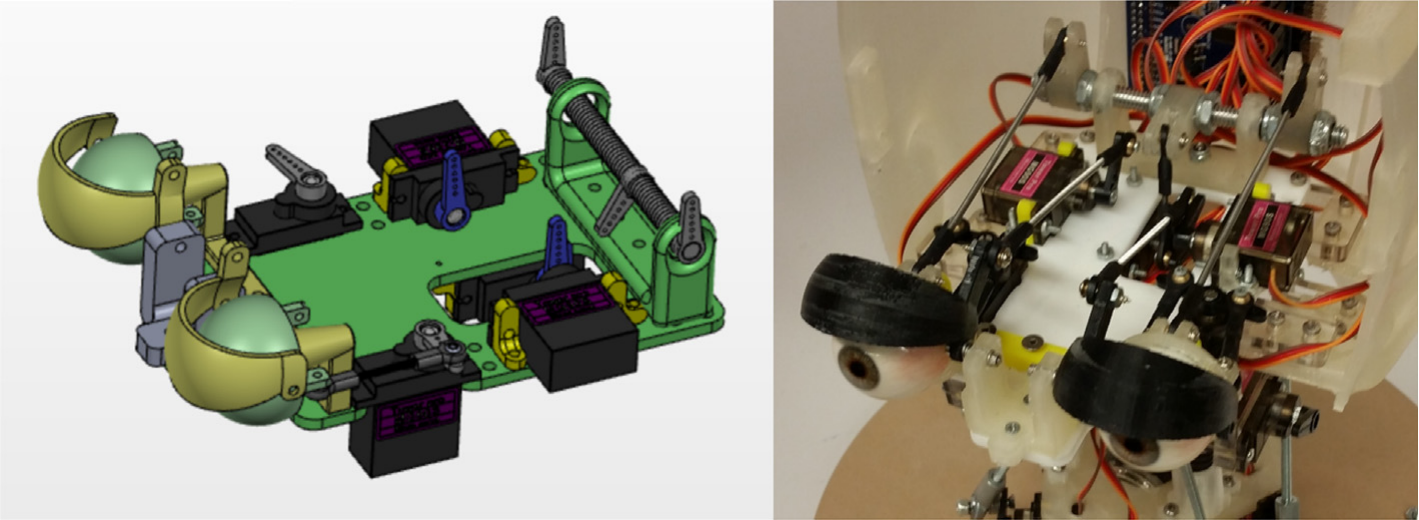
(left) Simplified CAD representation of eye mechanism, (right) final eye mechanism mounted in skull.

Fig. 6 shows a simplified CAD representation of the eye mechanism along with the final eye mechanism attached to the skull. A single servo motor is used to control the vertical rotation of both eyeballs. The horizontal rotation of the left and right eyeballs is independent of each other, with one servo horizontally rotating the left eyeball and another servo horizontally rotating the right eyeball. Similarly, the opening and closing of the eyelids is independent of each other, with one servo controlling the left eyelid and another servo controlling the right eyelid. A total of five servo motors are used in the eye mechanism.

**Fig. 7:**
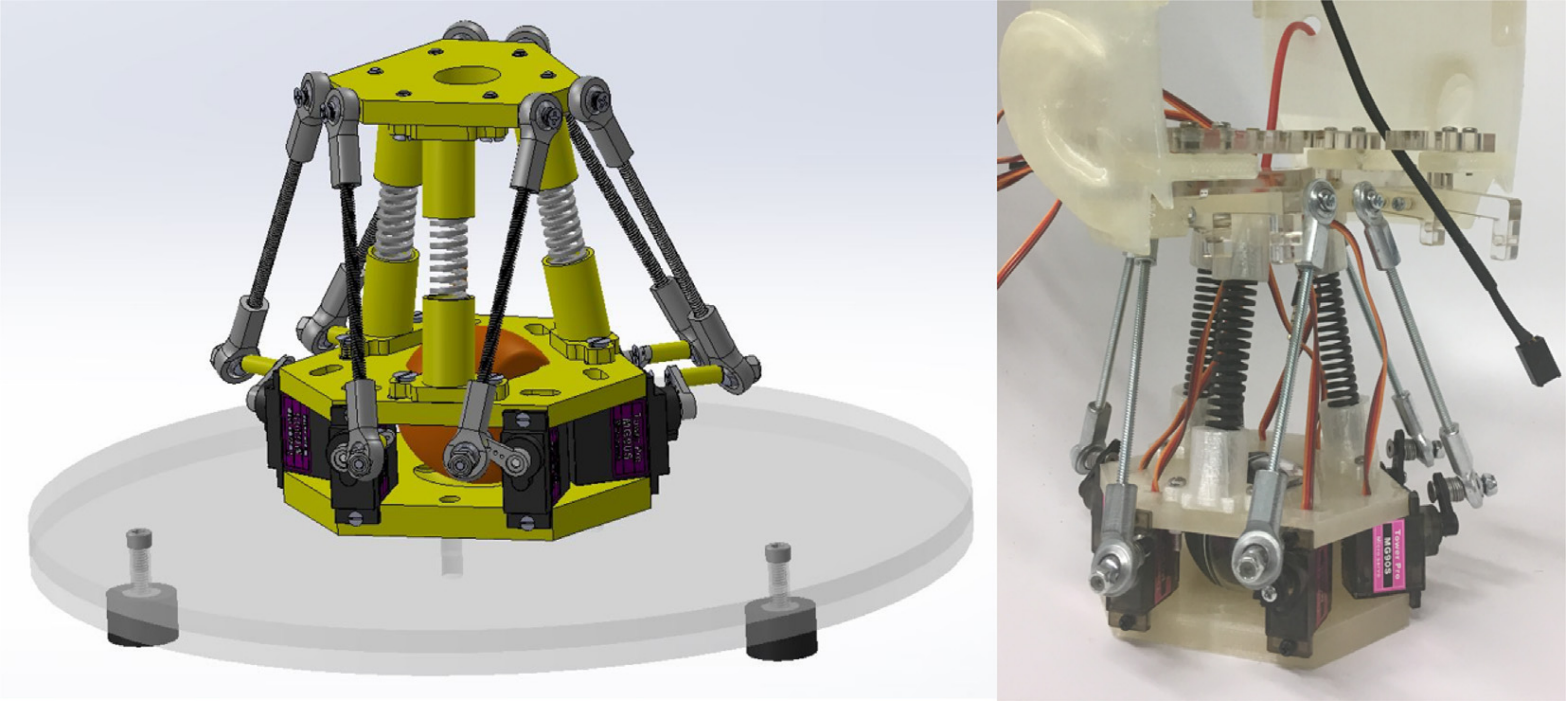
(left) CAD model of neck mechanism attached to base platform, (right) final neck mechanism attached to skull.

The neck mechanism shown in Fig. 7 is essentially a Stewart platform with some modifications to increase its load capacity. In particular, three load bearing springs are placed concentrically between the middle and top plates of the Stewart platform so that the weight of the head is mainly supported by the springs rather than by the servo motors. Without these springs, the servo motors would be unable to support the weight of the assembled head. Additionally, the neck mechanism is attached to an acrylic base platform with a relatively large diameter to prevent Eva from tipping over when it tilts its head forward. The rubber bumpers screwed into the bottom of the base platform provide a stable base and dampen vibrations during operation. Finally, a mini speaker is housed in the center between the bottom and middle plates to output audio from Eva’s text-to-speech software.

**Fig. 8:**
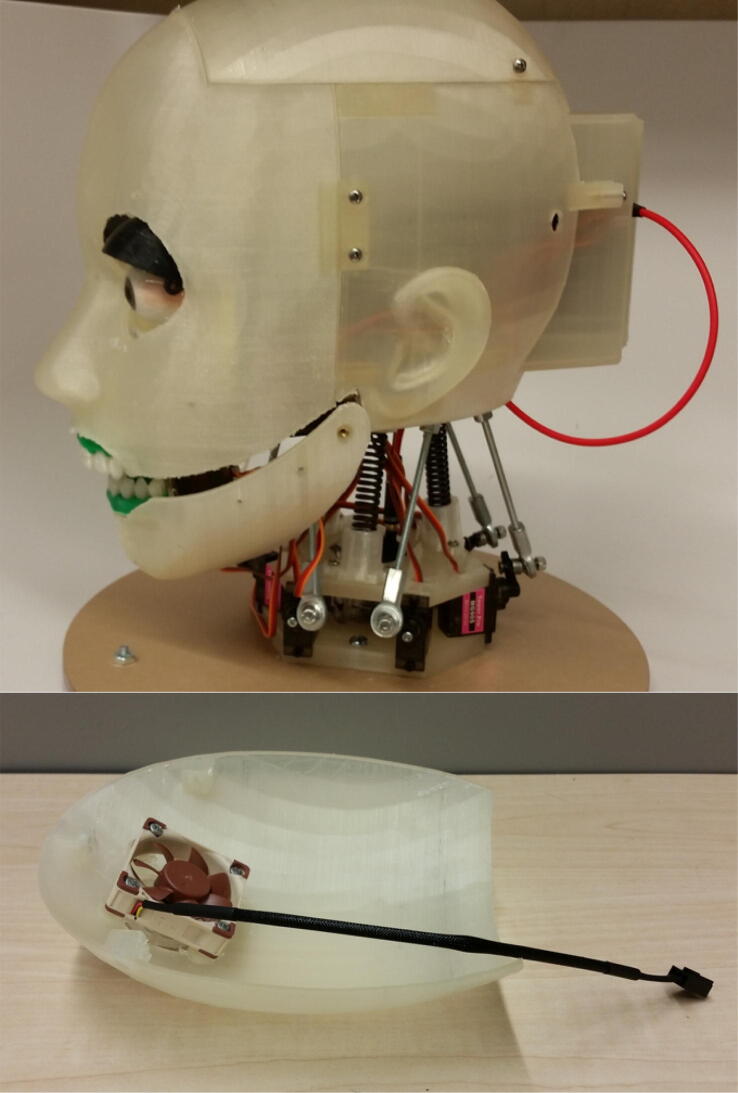
(top) 3D printed skull with all four pieces assembled, (bottom) cooling fan attached to top piece of skull.

An essential auxiliary component is the skull, which serves as a support for the silicone mask and an interface for all of the mechanisms. The skull is 3D printed in four pieces and attached together using heat-set inserts and 2–56, 4–40, and M2.5 screws. Fig. 8 shows the four pieces of the skull attached together. The first piece is the face which supports the silicone mask. The second piece is the back of the head which serves as the main attachment interface for all of the mechanisms and components. The third piece is the top of the head which houses the cooling fan. The fourth piece is the Raspberry Pi holder which houses the Raspberry Pi and servo hats. As the 3D printed jaw is part of the jaw mechanism and is not stationary, it is not considered part of the skull. Fig. 8 also shows that the 3D printed upper gums (green) and teeth (white) are attached to the face portion of the skull. Similarly, the lower gums and teeth are attached to the 3D printed jaw.

Another important auxiliary component is the cooling fan, which is attached to the top piece of the skull, as shown in Fig. 8. This fan, purchased from Noctua, was chosen because it is compact and exceptionally quiet. The fan ensures that the motors and electronics inside the skull do not overheat during regular operation of Eva.

**Fig. 9:**
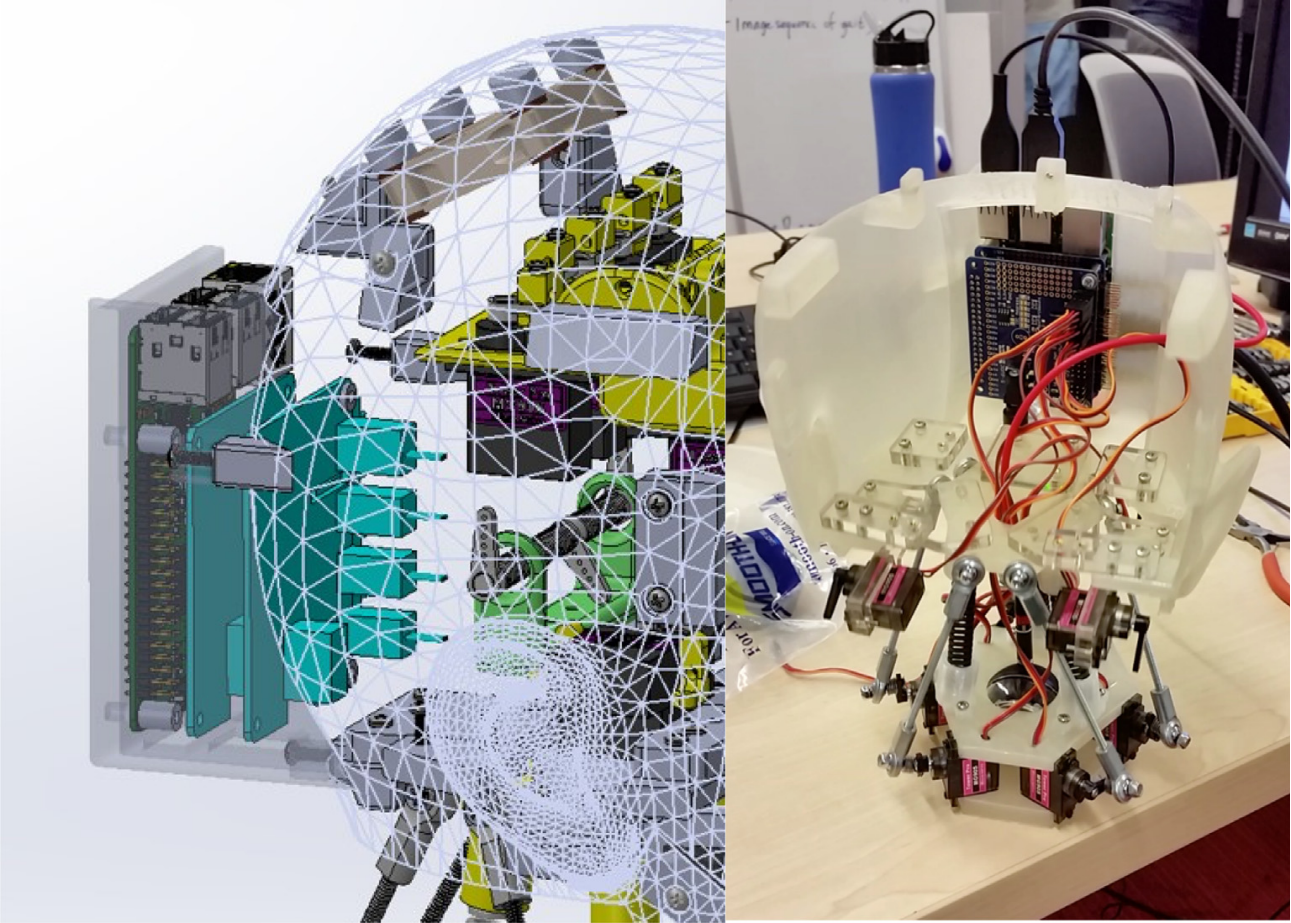
(left) CAD model of Raspberry Pi and servo hats in full assembly, (right) actual Raspberry Pi and servo hats attached to the Raspberry Pi holder.

The Raspberry Pi, which controls all of the servo motors and runs the Python code used to operate Eva, is attached to the Raspberry Pi holder, as shown in Fig. 9. The Raspbery Pi holder is a critical piece of the skull. Two 16 channel PWM servo hats are stacked on top of the Raspberry Pi’s connector pins, allowing the Raspberry Pi to control up to 32 servo motors. The Raspberry Pi is also used to connect the speaker via an aux cable, its own 2.5A power supply via a Micro USB cable, the 5 V 10A power supply for the servo motors and cooling fan via a 2.1 mm DC plug, the external monitor via an HMDI cable, and the external keyboard and mouse via two separate USB cables.

### 2.2 Utility for researchers

Eva’s usefulness for researchers can be summarized in four key points.

• Eva can be manufactured and assembled relatively inexpensively with accessible equipment. Other facially expressive robots have proprietary designs and the time and cost to build an alternative from scratch is far greater than the time and cost to build upon Eva’s current design.
• Eva can serve as a long-term platform for emotional AI research. Some organizations will offer their humanoid robots for rent but this is not a viable solution for conducting long-term academic research.
• Eva can be programmed in a multitude of languages, including Python, using open-source technologies on the Raspberry Pi. Eva’s functionality is not tied to the use of a companion app.
• Eva’s modular, open-source design allows for customization and improvements to its hardware. If documented and shared, these improvements will also aid other researchers in the field.

## 3 Design files

**Table:** 

Design file name	File type	Open source license	Location of the file
CAD Files.zip	CAD files	BSD 3-Clause	https://doi.org/10.5281/zenodo.3539723
Servo Code.zip	Python files	BSD 3-Clause	https://doi.org/10.5281/zenodo.3539723
Eva Demo.mp4	Video	BSD 3-Clause	https://doi.org/10.5281/zenodo.3539723
Servo-AU Correspondence	Figure	BSD 3-Clause	See Fig. 16
Emotion-AU Correspondence	Table	BSD 3-Clause	See Table 2

• CAD Files: contains the SolidWorks files for Eva and the molding rig used to create the silicone mask
• Servo Code: contains the sample Python files used to demonstrate Eva’s capabilities
• Eva Demo: a video demonstrating Eva’s capabilities
• Servo-AU Correspondence: a figure that details how the servos must actuate the silicone mask, jaw, and eyelids to express the six basic emotions
• Emotion-AU Correspondence: a table that details the FACS action units responsible for each of the six basic emotions

## 4 Bill of materials

**Table:** 

Designator	Component	Number	Cost per unit – USD	Total cost – USD	Source of materials	Material type
C1	CanaKit Raspberry Pi 3 Complete Starter Kit	1	74.99	74.99	https://www.amazon.com/dp/B01C6Q2GSY/ref=cm_sw_em_r_mt_dp_U_ZjYYDb58TFTV9	Non-specific
C2	Servo Hat for Raspberry Pi	2	17.50	35	https://www.adafruit.com/product/2327	Semiconductor
C3	Adafruit GPIO Stacking Header	1	7.11	7.11	https://www.amazon.com/dp/B00TW0W9HQ/ref=cm_sw_em_r_mt_dp_U_FrYYDb2XCJY8W	Metal, polymer
C4	Adafruit 3x4 Right Angle Male Headers	1	7.31	7.31	https://www.amazon.com/dp/B00NAY2894/ref=cm_sw_em_r_mt_dp_U_6sYYDb2MVNP3F	Metal, polymer
C5	Brass M2.5 Standoffs, Pack of 2	2	0.75	1.50	https://www.adafruit.com/product/2336	Metal
C6	MG90S Servo Motor	25	3.60	90	https://www.amazon.com/dp/B07FLXZ1VK/ref=cm_sw_em_r_mt_dp_U_EQYYDbFW93G82	Non-specific
C7	5 V 10A Switching Power Supply	1	29.95	29.95	https://www.adafruit.com/product/658	Non-specific
C8	Noctua Cooling Fan	1	13.95	13.95	https://www.amazon.com/dp/B00NEMGCIA/ref=cm_sw_em_r_mt_dp_U_bUYYDb18QJ5NA	Non-specific
C9	Leadsound Mini Speaker	1	16.99	16.99	https://www.amazon.com/dp/B01HB18IZ4/ref=cm_sw_em_r_mt_dp_U_3VYYDb9BVY5BR	Non-specific
C10	3.5 mm Male to Female Audio Extension Cable	1	7.99	7.99	https://www.amazon.com/dp/B01L5JZAEK/ref=cm_sw_em_r_mt_dp_U_C7YYDb9B1T34P	Non-specific
C11	Smooth-On Ecoflex 00-30	1	36.99	36.99	https://www.amazon.com/dp/B00CA5VY3U/ref=cm_sw_em_r_mt_dp_U_S8YYDbNTGYGN1	Polymer
C12	Smooth-On Silc-Pig Silicone Pigment	1	27.98	27.98	https://www.amazon.com/dp/B005ZH0SFU/ref=cm_sw_em_r_mt_dp_U_d-YYDb7KJK9P7	Inorganic
C13	Smooth-On Universal Mold Release	1	20.80	20.80	https://www.amazon.com/dp/B004BNHLOK/ref=cm_sw_em_r_mt_dp_U_Y.YYDbWW87VRQ	Inorganic
C14	Green Eyes	1	40.00	40.00	http://www.tech-optics.com/product/REV29	Polymer, Wood
C15	Ardell Deluxe Pack Lash, 120	1	5.68	5.68	https://www.amazon.com/dp/B00SX1V2T6/ref=cm_sw_em_r_mt_dp_U_vcZYDbA5K14T1	Inorganic
C16	Blink Black Eyebrow Extension	1	15.95	15.95	https://www.amazon.com/dp/B00Q8IBKV6/ref=cm_sw_em_r_mt_dp_U_tdZYDbQ2WX3GK	Inorganic
C17	Soft Flex 0.019′' Beading Wire	1	17.08	17.08	https://www.amazon.com/dp/B005DJV6GA/ref=cm_sw_em_r_mt_dp_U_nfZYDb4AH3T2X	Metal
C18	25ft Teflon Tubing	1	25.00	25.00	https://www.mcmaster.com/5239 k23	Polymer
C19	Compression Spring	3	3.16	9.48	https://www.mcmaster.com/9620 k11	Metal
C20	Rubber Bumper	3	6.28	18.84	https://www.mcmaster.com/2572 k9	Polymer
C21	Great Planes Pro Thread Locking Compound	1	6.36	6.36	https://www.amazon.com/dp/B001BHLSF2/ref=cm_sw_em_r_mt_dp_U_0jZYDb6KKT3N1	Inorganic
C22	Servo Linkage Stoppers, Pack of 10	2	7.61	15.22	https://www.amazon.com/dp/B01LBJKIZU/ref=cm_sw_em_r_mt_dp_U_nlZYDbYGE24BE	Metal
C23	Steel Ball Joint Rod End	12	6.26	75.12	https://www.mcmaster.com/60645k78	Metal
C24	M2 Ball Joints, Pack of 20	1	11.99	11.99	https://www.amazon.com/20pcs-Joints-Standard-Airplane-Replacement/dp/B01KSZ4L56/ref=sr_1_20?ie=UTF8&qid=1486492034&sr=8–20-spons&keywords=ball + joint + rod + end + rc&psc=1&pldnSite=1	Metal, Polymer
C25	0.25′' Steel Ball Bearing	2	6.00	12.00	https://www.mcmaster.com/57155k355	Metal
C26	2–56 Heat-set Inserts, Pack of 100	1	10.51	10.51	https://www.mcmaster.com/93365a110	Metal
C27	4–40 Heat-set Inserts, Pack of 100	1	11.19	11.19	https://www.mcmaster.com/93365a120	Metal
C28	M2.5 Heat Set Inserts, Pack of 100	1	11.19	11.19	https://www.mcmaster.com/94180a321	Metal
C29	1–64 Screws, Pack of 50	1	9.96	9.96	https://www.mcmaster.com/91771a176	Metal
C30	2–56 Shoulder Screw	2	5.21	10.42	https://www.mcmaster.com/99154a313	Metal
C31	2–56 × 0.25′' Screws, Pack of 100	1	3.00	3.00	https://www.mcmaster.com/90272a077	Metal
C32	4–40 × 0.5′' Screws, Pack of 100	1	1.80	1.80	https://www.mcmaster.com/90272a110	Metal
C33	M2.5, 14 mm Long Screws, Pack of 100	1	4.34	4.34	https://www.mcmaster.com/92000a108	Metal
C34	5–40 × 0.75′' Flat Head Screws, Pack of 25	1	2.75	2.75	https://www.mcmaster.com/92210a131	Metal
C35	8–32 × 0.75′' Screws, Pack of 100	1	4.22	4.22	https://www.mcmaster.com/90272a197	Metal
C36	1–64 Hex Nut, Pack of 100	1	8.10	8.10	https://www.mcmaster.com/91841a038	Metal
C37	2–56 Locknuts, Pack of 5	1	12.33	12.33	https://www.mcmaster.com/95307a800	Metal
C38	5–40 Locknuts, Pack of 100	1	2.88	2.88	https://www.mcmaster.com/90631a006	Metal
C39	1/4′'-20 Lock Nuts, Pack of 100	1	3.71	3.71	https://www.mcmaster.com/90566a029	Metal
C40	M2 Fully Threaded Rod	5	4.23	21.15	https://www.mcmaster.com/90024a210	Metal
C41	Steel 6–32 Threaded Studs, Pack of 10	1	2.88	2.88	https://www.mcmaster.com/95475a238	Metal
C42	Aluminum 1/4′'-20 Threaded Studs, Pack of 10	1	14.82	14.82	https://www.mcmaster.com/93225a882	Metal

Eva was designed to be manufactured for approximately $900 with access to standard tools, a laser cutter, and a desktop 3D printer. The current prototype was printed on an Ultimaker 2 + Extended but any desktop 3D printer can be used as long as it has a build height of at least 11 in.. Approximately 2.25 kg of 3D printing material (ABS or PLA) will be needed, at an approximate cost of $120. Note that PLA was used for all of the 3D printed parts in the current prototype.

## 5 Build instructions

### 5.1 Construction of components

Due to Eva’s modular design, its various components can be constructed independently before later being assembled together. The main components that must be constructed are the neck mechanism, the eye mechanism, the jaw mechanism, and the mask actuation mechanism. Important auxiliary components that must be constructed include the skull, the silicone mask, and the base platform. Eva also makes use of several off-the-shelf components such as the Raspberry Pi, Adafruit servo hats, MG90S servo motors, Noctua cooling fan, and Leadsound mini speaker, which do not need to be modified.

**Fig. 10:**
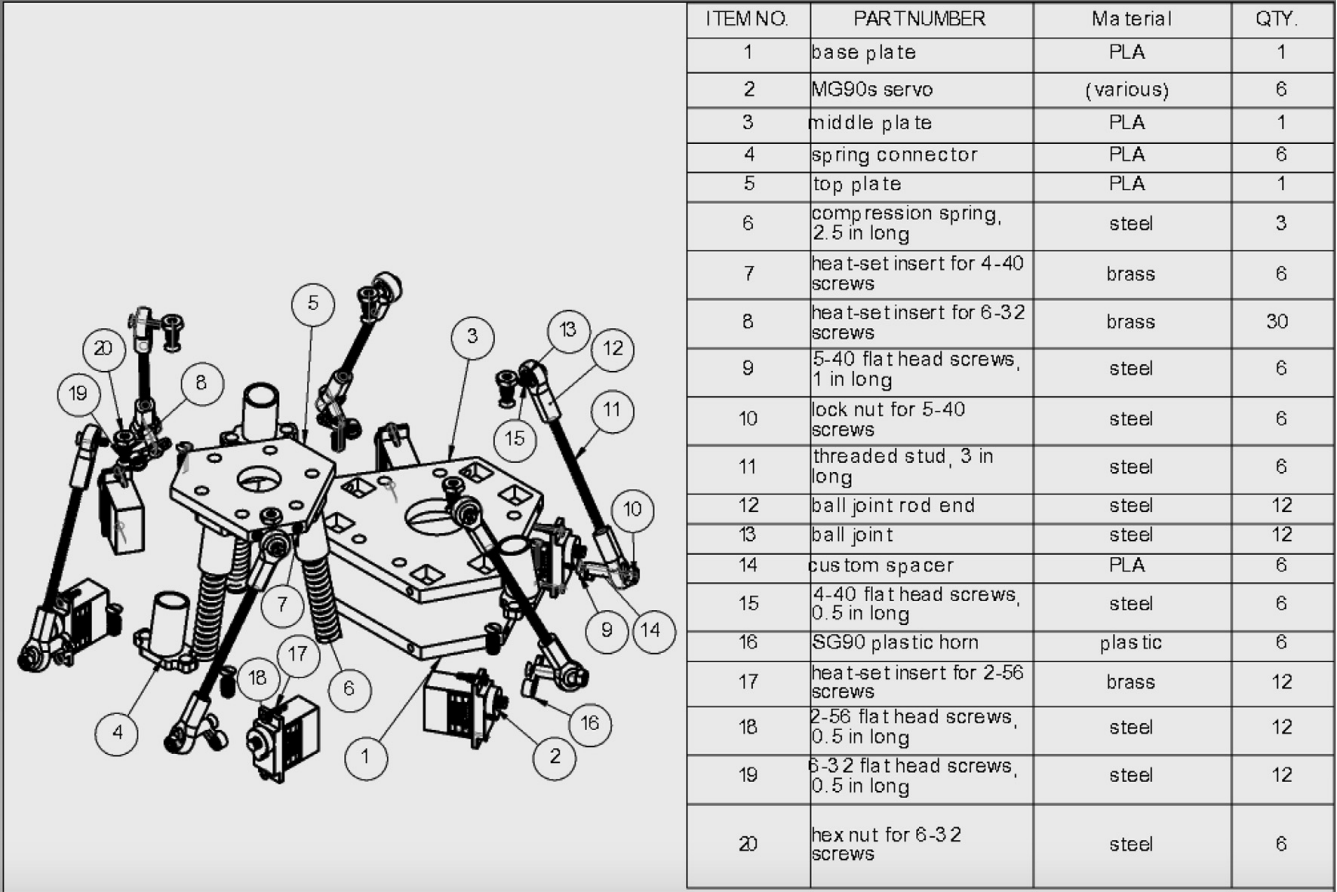
Exploded view of the neck mechanism with the corresponding bill of materials.

The neck mechanism shown in Fig. 7 consists of three 3D printed plates, six 3D printed spring connectors, six 3D printed spacers, six MG90S servos, three compression springs, and various screws, heat-set inserts, and ball joints. A complete breakdown of the neck mechanism’s components can be found in Fig. 10. For detailed information on the construction of the neck mechanism, open the neck mechanism assembly file within the “CAD Files.zip” directory referenced in Section 3. In broad terms, all 3D printed parts should first be printed, with heat-set inserts installed in the designated locations and the spring connectors attached to the middle and top plates. The servos should then be attached to the bottom and middle plates with the appropriate screws, making sure to place the speaker in between the bottom and middle plates in its designated cavity. The three springs should then be placed in the cavity of the spring connectors and all ball joints and ball rod ends should be attached to the servos. Finally, position the top plate so that the springs fit inside the spring connectors of the top plate and attach the ball joints to the top plate with the appropriate screws.

As shown in Fig. 7, the acrylic base platform is an auxiliary component of the neck mechanism, designed to provide Eva with a wide and stable base to prevent tipping when the head tilts forward. For detailed information about the acrylic base platform, open the base platform assembly file within the “CAD Files.zip” directory referenced in Section 3. To construct the base platform, the two constituent acrylic plates should first be laser cut, the heat-set inserts then installed, and the rubber bumpers finally attached. To simplify the assembly process, the bottom plate of the neck mechanism should first be attached via screws to the acrylic base platform before attaching the servos.

The jaw mechanism, as depicted in Fig. 4, is relatively simple, consisting of two MG90S servo motors, a 3D printed jaw, 3D printed lower gums and lower teeth, and heat-set inserts with their corresponding screws. The jaw also houses the bottom servo bank used in the mask actuation mechanism, which consists of a 3D printed frame, three MG90S servos, and heat-set inserts with their corresponding screws. For detailed information about the jaw mechanism, open the jaw mechanism assembly file within the “CAD Files.zip” directory referenced in Section 3. To construct the jaw mechanism, first 3D print the jaw, the lower gums and teeth, and the frame for the bottom servo bank. Proceed by installing all heat-set inserts into the 3D printed parts as indicated in the CAD jaw assembly. Next, attach the lower gums and teeth and the two servo motors to the jaw with screws. Finally, attach the three servo motors to the bottom servo bank frame with screws and screw the entire bottom servo bank to the 3D printed jaw.

**Fig. 11:**
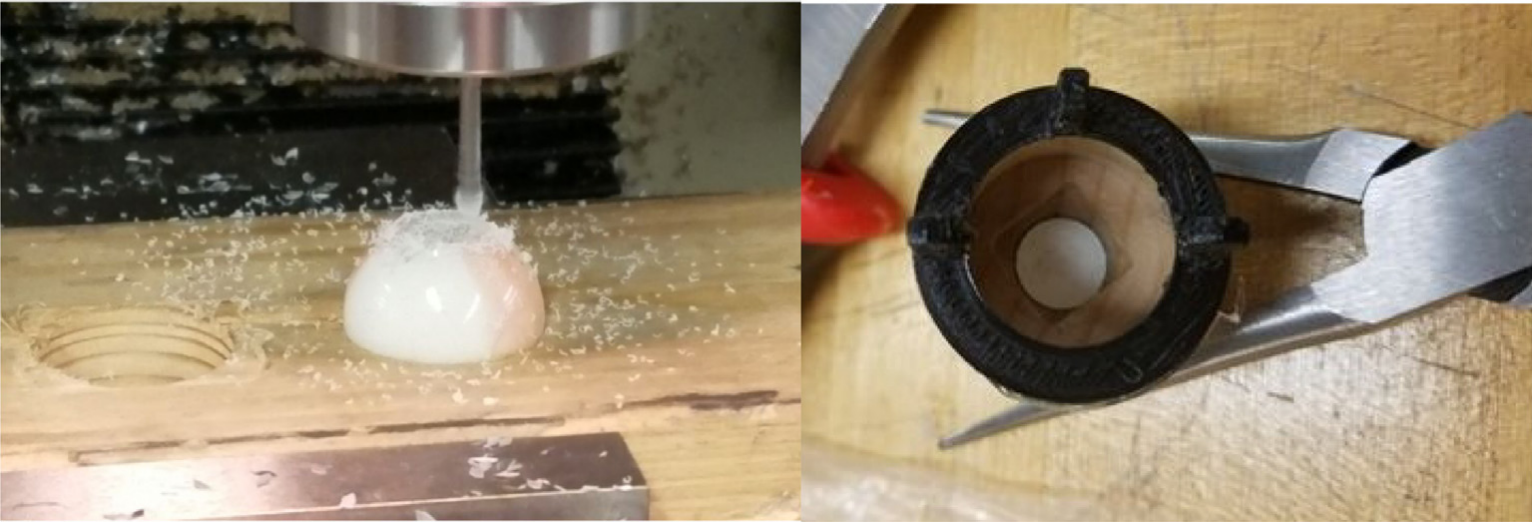
(left) Machining an eyeball on a milling machine by press fitting the eyeball into a hole to secure it, (right) attaching the 3D printed attachment to the machined eyeball.

Building the eye mechanism shown in Fig. 6 requires the use of a 3D printer, a laser cutter, and a milling machine. The eyelids, the eyeball joints, and the various attachment parts must be 3D printed while the acrylic baseplate must be laser cut. Refer to the eye mechanism assembly in “CAD Files.zip” for all of the relevant design files. The eyeballs are the only parts in the full Eva assembly that need to be machined. As demonstrated in Fig. 11, the core of each eyeball must be machined out to provide an interface for the 3D printed ball joints. The eyeballs can be secured during the milling process by press fitting them into a hole in a block of wood and then securing that block in the mill’s vice. Once the eyeballs are machined, the 3D printed attachments that interface with the ball rods and ball rod ends can be snapped onto the outer ring of the eyeballs. Once the custom parts have been manufactured, all components should be attached to the acrylic baseplate with screws as indicated in the eye mechanism assembly file.

In addition to the bottom servo bank contained in the jaw, a top servo bank assembly must also be built for the mask actuation mechanism, as depicted in Fig. 2. This assembly consists of a 3D printed frame, nine servo motors, nine servo linkage stoppers, and heat-set inserts with their corresponding screws. The design files for this assembly can be found in the servo bank assembly file contained in the “CAD Files.zip” directory referenced in Section 3. Note that the CAD assembly indicates the presence of ten servo motors when only nine are used in the final assembly. The tenth motor is for the actuation of an AU that was found to be nonessential, as explained in Section 5.4. Once the frame has been 3D printed and all heat-set inserts have been installed, the servo motors can be attached with screws. The linkage stoppers can then be attached to the servo horns with screws.

**Fig. 12:**
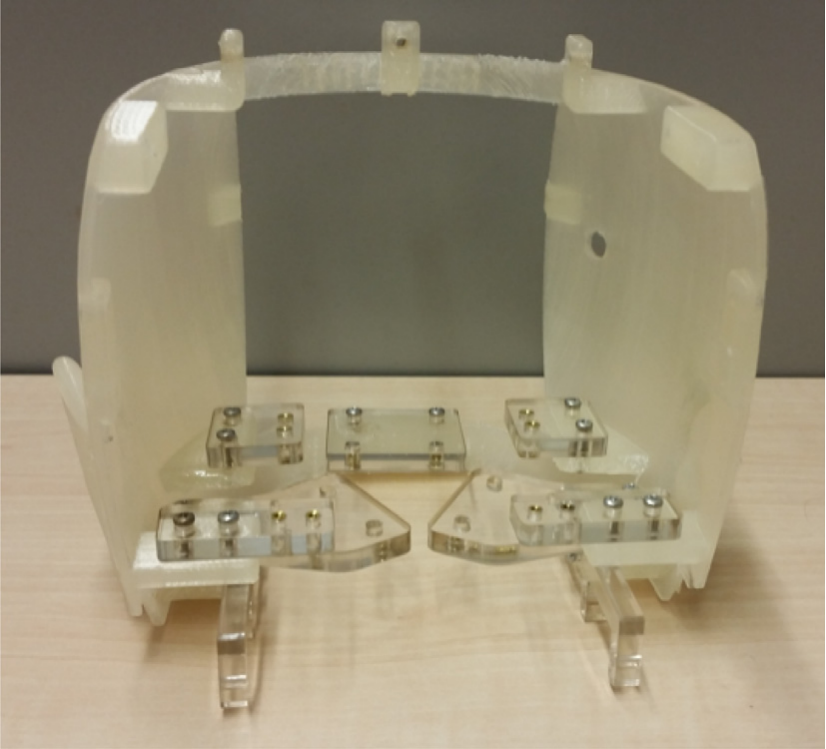
Skull (back) assembly with acrylic attachments that serve as interfaces for the other mechanisms.

A critical auxiliary component that needs to be manufactured is the skull. As described in Section 2.1 and illustrated in Fig. 8, the skull is divided into four pieces: the face, the back, the top, and the Raspberry Pi holder. Each of these pieces needs to be 3D printed separately. The skull (back) assembly also utilizes several acrylic attachments that must be laser cut, as shown in Fig. 12. These acrylic attachments serve as the interface for most of Eva’s other components. The skull (back) and the acrylic attachments heavily make use of heat-set inserts and the attachments are secured to the skull (back) with screws. Similarly, heat-set inserts and screws are used to attach the cooling fan to the skull (top) and the upper gums and teeth to the skull (face), as shown in Fig. 8. The Raspberry Pi is also attached to the Raspberry Pi holder with heat-set inserts and screws, as shown in Fig. 9. The design files to construct the skull can be found in the “CAD Files.zip” directory referenced in Section 3.

**Fig. 13:**
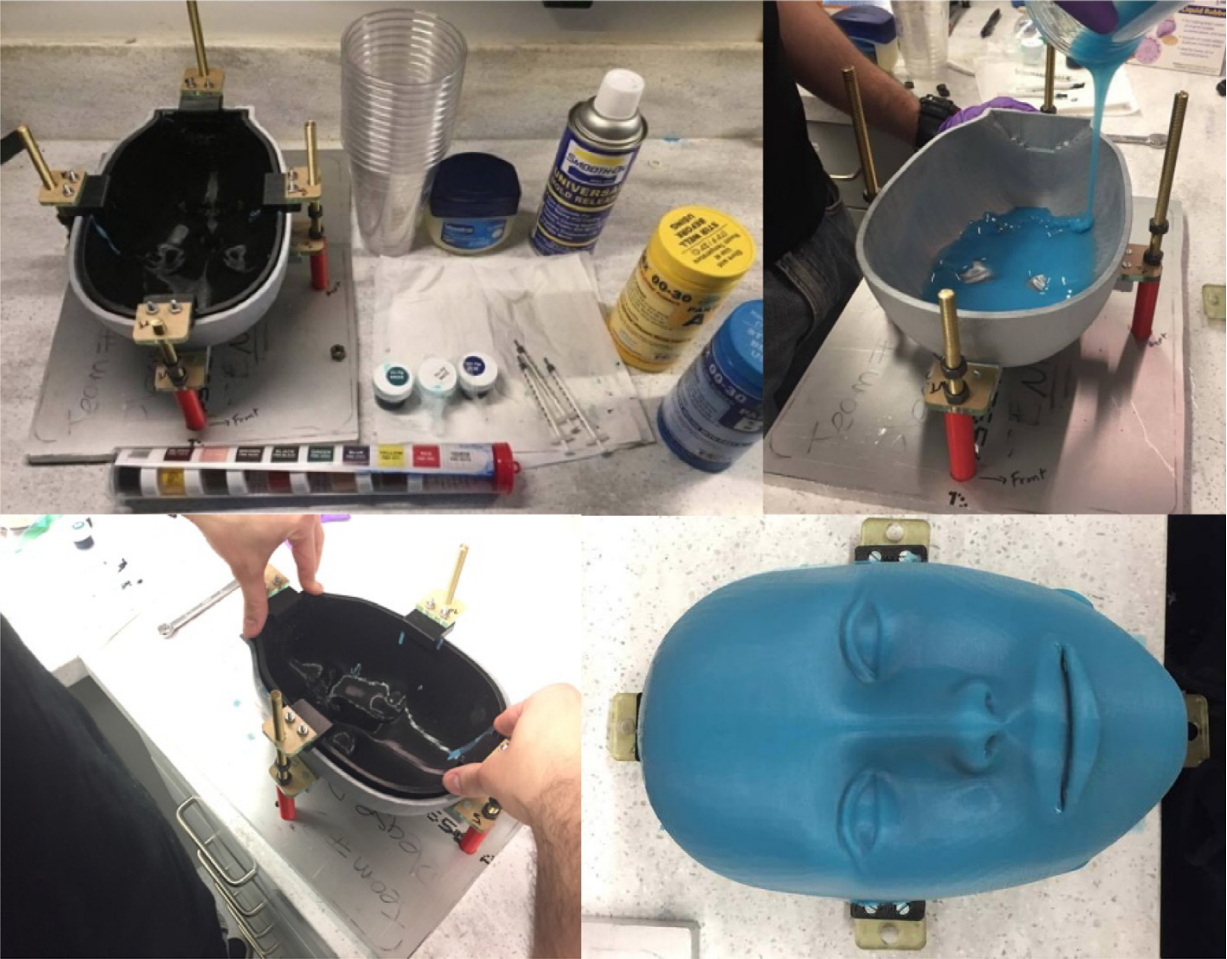
Silicone mask casting process: (upper left) setup and materials, (upper right) pouring dyed silicone into negative mold, (lower left) pressing the positive mold into the negative mold, (lower right) mask after removing positive mold.

The last critical component that must be manufactured is the silicone mask. Fig. 13 provides a visual summary of the casting process utilized to manufacture the mask. The process utilizes 3D printed positive and negative molds that are identical to the outer surface of the skull (face) and jaw. The negative mold is slightly larger than the positive mold so that the mask is about 0.08in thick. The setup also requires a machined aluminum base plate, threaded rods, laser-cut acrylic attachments, and nuts for pressing the molds together. The mask is made from Smooth-On EcoFlex 00-30, a type of silicone that closely mimics the material properties of human skin. The silicone is died blue with Smooth-On Silc-Pig and Smooth-On Universal Mold Release is sprayed on the inner surface of the negative mold to aid in separation of the molds after curing. With the positive mold removed, the mixed silicone is then poured into the negative mold and moved around so that it covers most of the negative mold. The positive mold is then placed into the negative mold and pressed down by tightening the nuts threaded into the threaded rods above the acrylic attachments of the positive mold. After waiting at least four hours for the silicone to cure, the nuts are released and the positive mold is separated from the negative mold, leaving the cured silicone mask on the surface of the positive mold. Complete information about this setup can be found by referring to the molding rig assembly file in the “CAD Files.zip” directory referenced in Section 3.

### 5.2 Assembly of components

**Fig. 14:**
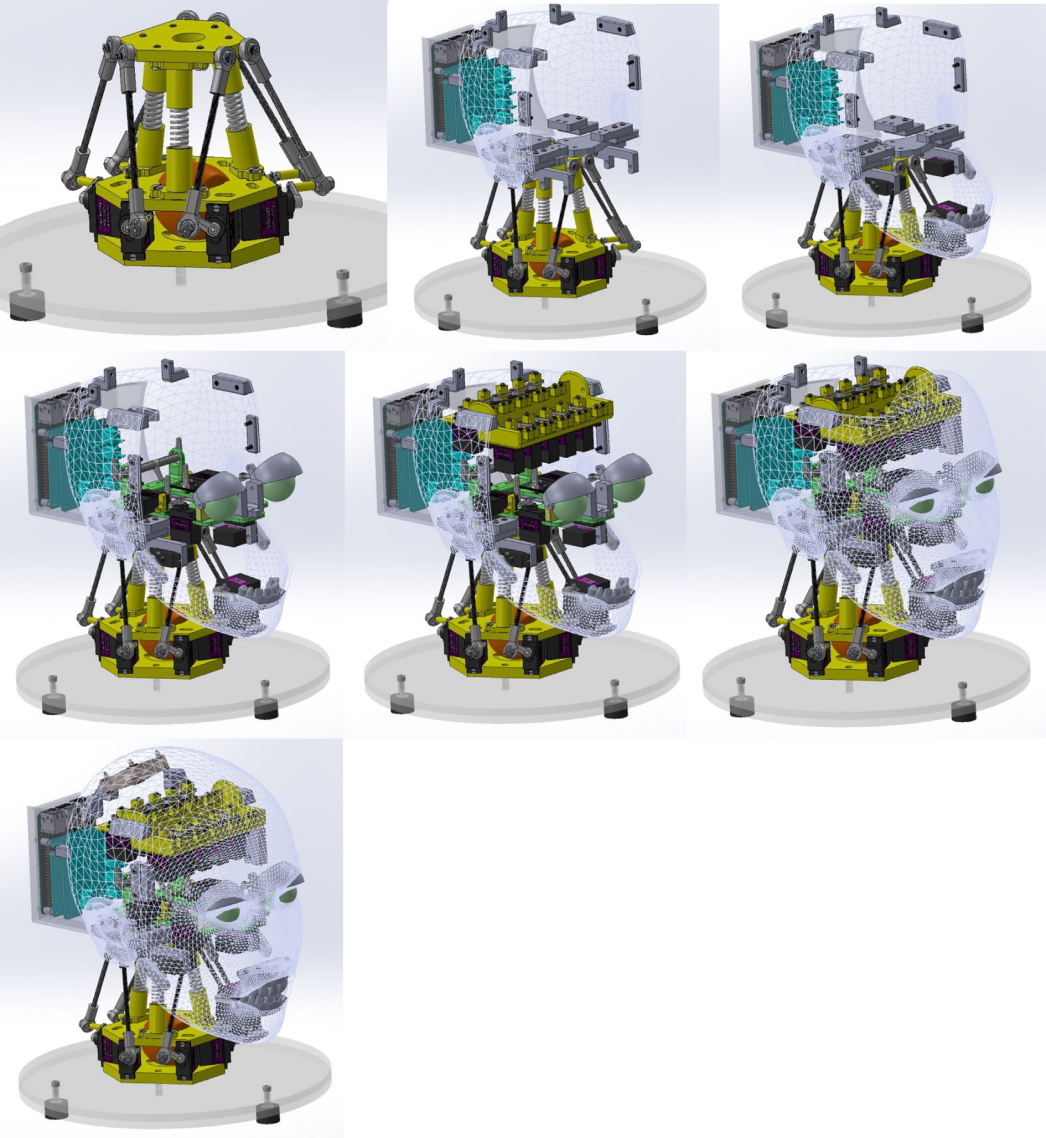
Eva’s assembly sequence (ordered left to right, top to bottom).

Due to the large number of components that must be fit within the skull, it is imperative that the components be assembled in a particular sequence. This sequence is illustrated in Fig. 14. Starting with the assembled neck mechanism attached to the base platform, the skull (back) and Raspberry Pi holder assembly must be attached to the top plate of the neck mechanism with screws at the specified interface locations. The interface locations for all of the components and the screws used for attachment can be determined by opening the full assembly file in the “CAD Files.zip” directory specified in Section 3. Next, the assembled jaw mechanism must be attached with screws to the skull (back) at the specified interface points near the ears. The assembled eye mechanism must then be placed onto the interfaces of the skull (back) and secured with screws. Then, the top servo bank must be secured with screws to the skull (back) at the specified interfaces near the top of the head. The skull (face) assembly should then be attached with screws to the skull (back) interfaces on the sides of the head. Before attaching the skull (top) assembly, the silicone mask must first be installed as depicted in Fig. 3. The mask is installed by inserting each steel wire through its corresponding hole in the skull (face), feeding the wire through the corresponding Teflon tube, and attaching the wire with a screw to its corresponding servo motor via a linkage stopper on the servo horn. To determine which tube corresponds to which wire, refer to Fig. 16. It is essential that the wires be feed from bottom to top, i.e., the wires near the jaw of the mask must be inserted first and the wires near the eyes must be inserted last. Finally, once all of the wires are secured to the servo motors and the mask is resting firmly against the skull (face), the skull (top) assembly can be attached to the skull (back) with screws at the interfaces at the top of the head.

### 5.3 Potential construction and assembly hazards

The construction and assembly of Eva primarily relies on the use of a 3D printer, a laser cutter, and standard hand tools like screwdrivers and pliers, so the potential hazards in this process are limited. However, machining the eyeballs and the aluminum plate used in the molding rig does require the operation of a milling machine and caution should always be taken whenever operating heavy machinery. When operating a laser cutter, it is also critical to follow all safety procedures and ensure that fumes are properly ventilated. As assembling Eva requires one to place their hands in the small confines of the skull, it may also be prudent to wear light gloves to avoid abrasions from accidental contact with the inner components.

### 5.4 Design decisions and prototypes

**Fig. 15:**
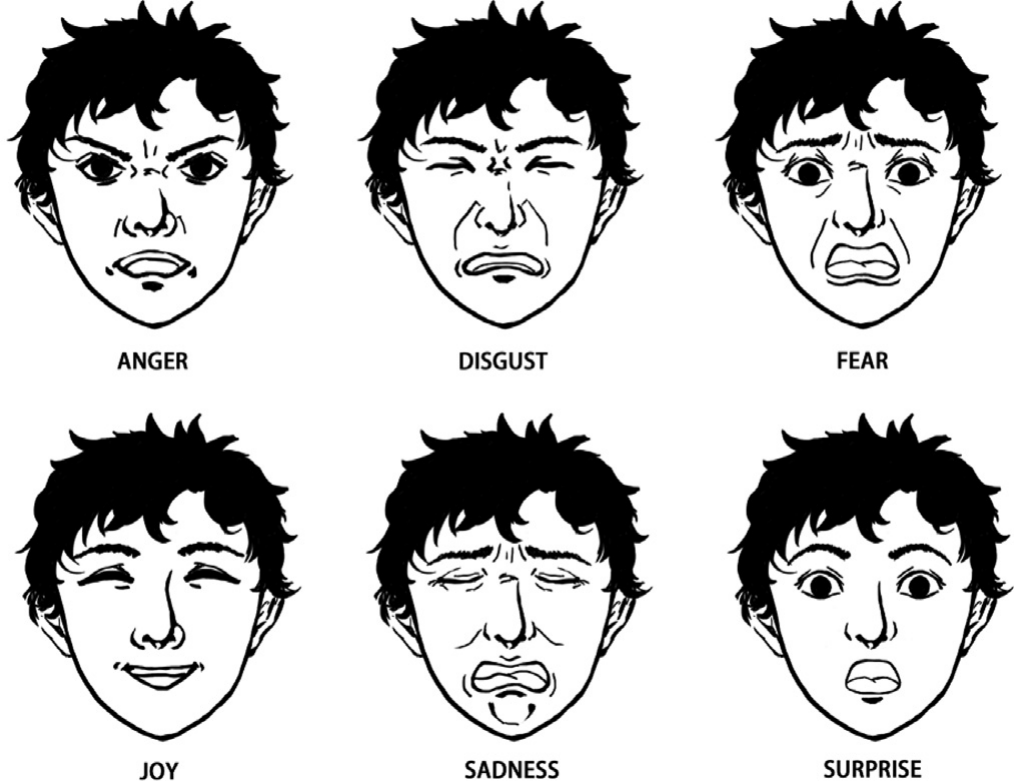
The six basic emotions hypothesized by Paul Ekman (image credit: Boyuan Chen).

One of the most important aspects of the design process was determining how to actuate the silicone mask to emulate human facial expressions. This part of the design relied on the Facial Action Coding System formulated by Paul Ekman [13]. According to Paul Ekman, a renowned psychologist and a pioneer in the study of facial expressions, all facial expressions can be linked to six basic emotions [14]. These emotions are anger, disgust, fear, joy, sadness, and surprise, as illustrated in Fig. 15. Thus, Eva’s design is grounded in these six basic emotions. Note that the facial expression corresponding to each of these six emotions is characterized by the unique contraction of certain facial muscles.

Eva is designed to generate a wide range of human facial expressions by utilizing one or more of these six basic emotions. For example, while joy would correspond to one facial expression, the combination of joy and surprise would result in happily surprised, which would correspond to a separate facial expression. Not all emotional combinations will result in a comprehensible facial expression, but the list of reproducible facial expressions can generally be expanded by combining certain emotions.

As previously mentioned, each emotion corresponds to a specific movement of the facial muscles. For example, joy corresponds to an upward and inward movement of the corners of the mouth while surprise corresponds to an upward movement of the eyebrows. Combined emotions correspond to a combination of the individual facial movements associated with each emotion. For example, happily surprised is expressed by an upward and inward movement of the corners of the mouth as well as an upward movement of the eyebrows. While these two facial muscle contractions are the most obvious and well-known indicators of these two emotions, in reality, there are two to six unique muscle contractions responsible for producing each of these six basic facial expressions. Due to the complexity of facial muscle anatomy, it is necessary to develop a formal system for analyzing these muscle contractions.

The Facial Action Coding System developed by Paul Ekman builds upon the six-emotion framework by cataloging all of the facial movements capable of being performed by humans and identifying which facial movements correspond to which facial expressions. FACS is based on the concept of an Action Unit (AU), or the contraction or relaxation of one or more facial muscles. There are a total of 46 distinct AUs which are responsible for generating all human facial expressions.

**Fig. 16:**
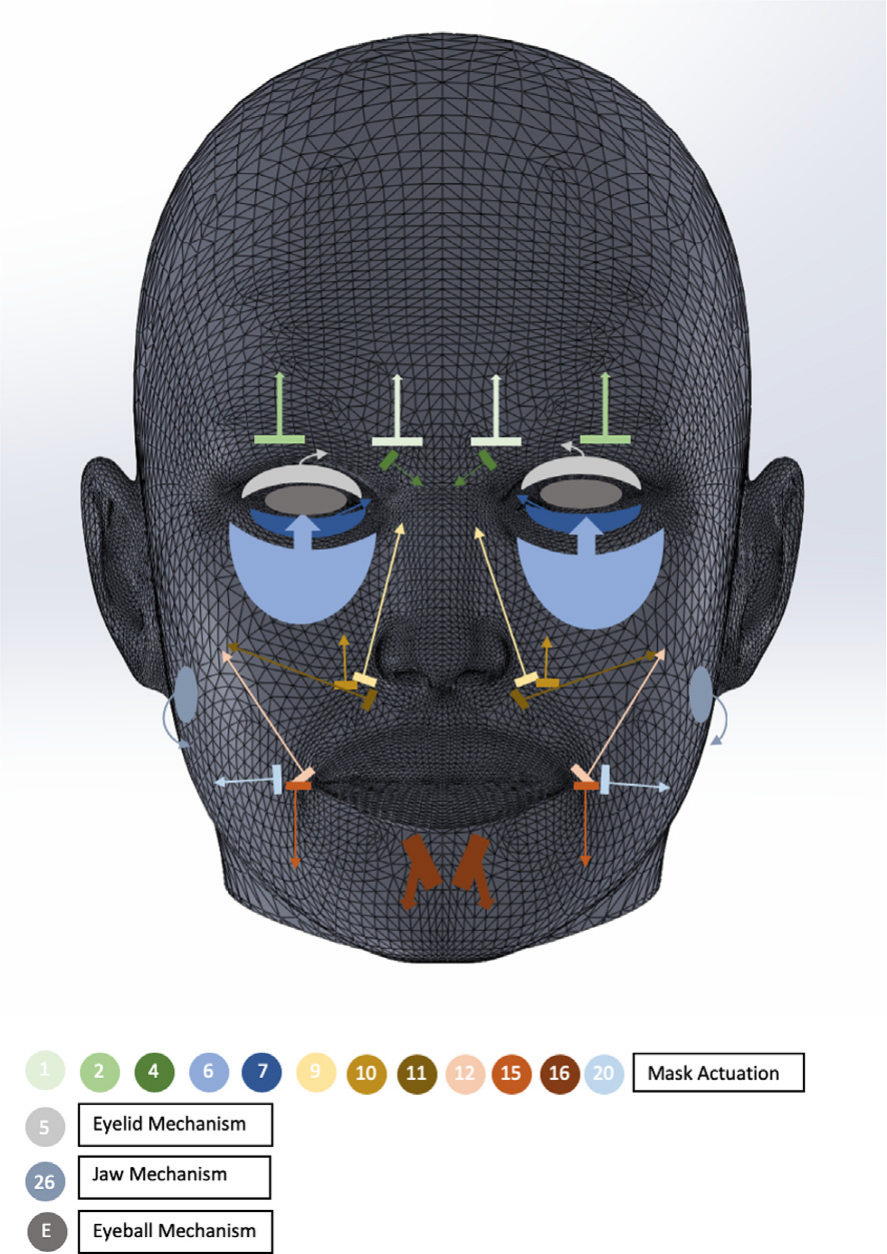
Graphical representation of all AUs used to generate Eva’s facial expressions.

By employing FACS, it was relatively simple to identify which AUs were necessary to produce each basic expression, and then actuate the mask to reproduce these AUs. For example, joy can be reproduced from AUs 6 and 12 while surprise can be reproduced from AUs 1, 2, 5, and 26. Fig. 16. graphically details how the mask is actuated to reproduce each of these AUs. Note that in order to minimize the complexity of the system, only the essential AUs for each expression have been employed, resulting in a total of 12 separate AUs to actuate the mask. An additional three AUs corresponding to the eyelids, jaw, and eyeballs are also utilized.

For clarity, Table 2 details which AUs are utilized to reproduce each of the six basic emotions.

**Table 2:** Emotion-AU correspondence (specifies the AUs used to express each emotion).

Emotion	Action Units
Surprise	(1 + 2 + 5 + 26)
Fear	(1 + 2 + 4 + 5 + 20 + 26)
Joy	(6 + 12)
Sadness	(1 + 4 + 11 + 15)
Disgust	(9 + 15 + 16 + 26)
Anger	(4 + 5 + 7 + 10 + 26)

**Fig. 17:**
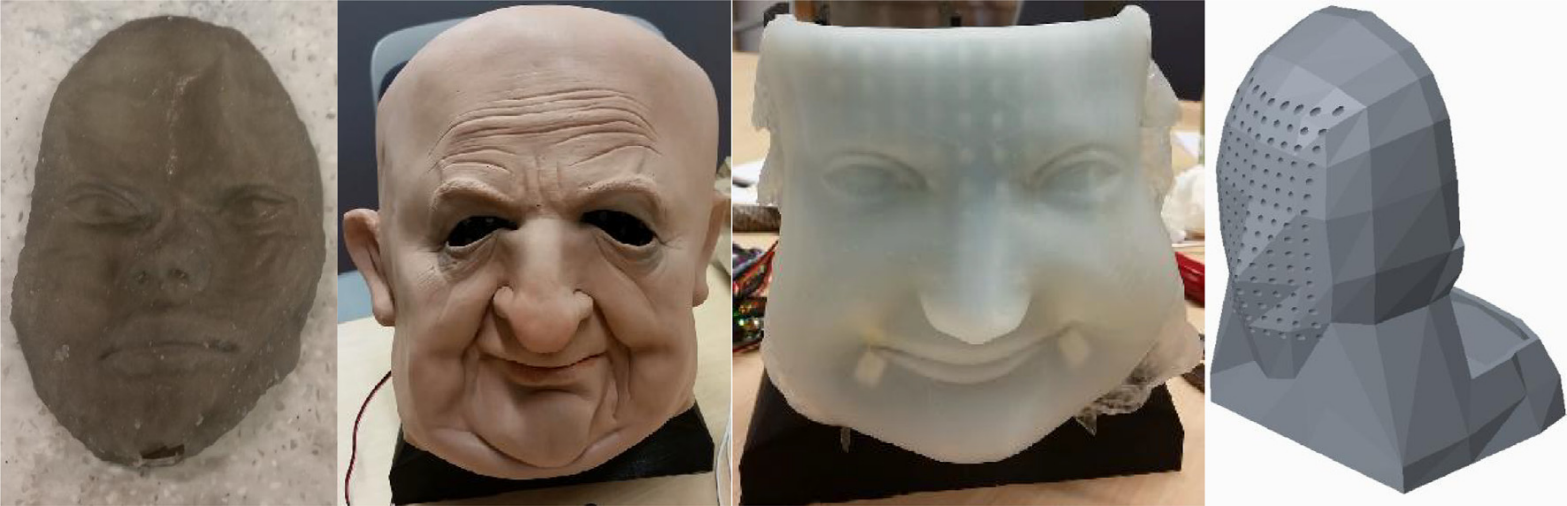
From left to right: (1) mask 3D printed from TangoBlack+, (2) happy expression with off-the-shelf latex mask on generic skull, (3) happy expression with silicone mask on generic skull, (4) 3D printed generic skull.

The final design for the mask actuation mechanism was informed by the shortcomings of several early prototypes. For instance, two other mask materials, TangoBlack + and latex, were tested before settling on silicone, as shown in Fig. 17. While both TangoBlack + and latex can mimic the surface of the human face reasonably well, their material properties are highly different than that of human skin. These materials are far too rigid and cannot be properly actuated to emulate facial expressions. Thus, despite the difficulty of casting a silicone mask, silicone was chosen as the mask material because of its ability to closely emulate the properties of skin.

Another prototype that greatly influenced the final design for Eva was a prototype of the skull, also shown in Fig. 17. This skull generally followed the contours of the human face but its outer surface was not designed to match that of a specific mask. As a result, actuating a mask on the skull caused the mask to cave inwards because of the presence of gaps between the outer surface of the skull and the inner surface of the mask. This result was replicated for both a latex and a silicone mask, as seen in Fig. 17, so the issue was clearly with the interface between the skull and mask rather than the properties of the mask. To solve this issue, the final design for Eva utilizes a skull and mask that are manufactured based on the same profile of a human face.

The considerable time and cost of 3D printing this skull also revealed the need for a more modular design, so that small changes to the design of the skull did not necessitate another full print. This insight resulted in the four skull pieces and jaw present in Eva’s final design. Laser-cut acrylic attachments were also used to serve as the interface between components so that changes to the size or location of one component did not affect the design of linked components. New acrylic attachments can be easily and precisely laser cut to create a new interface between modified components.

**Fig. 18:**
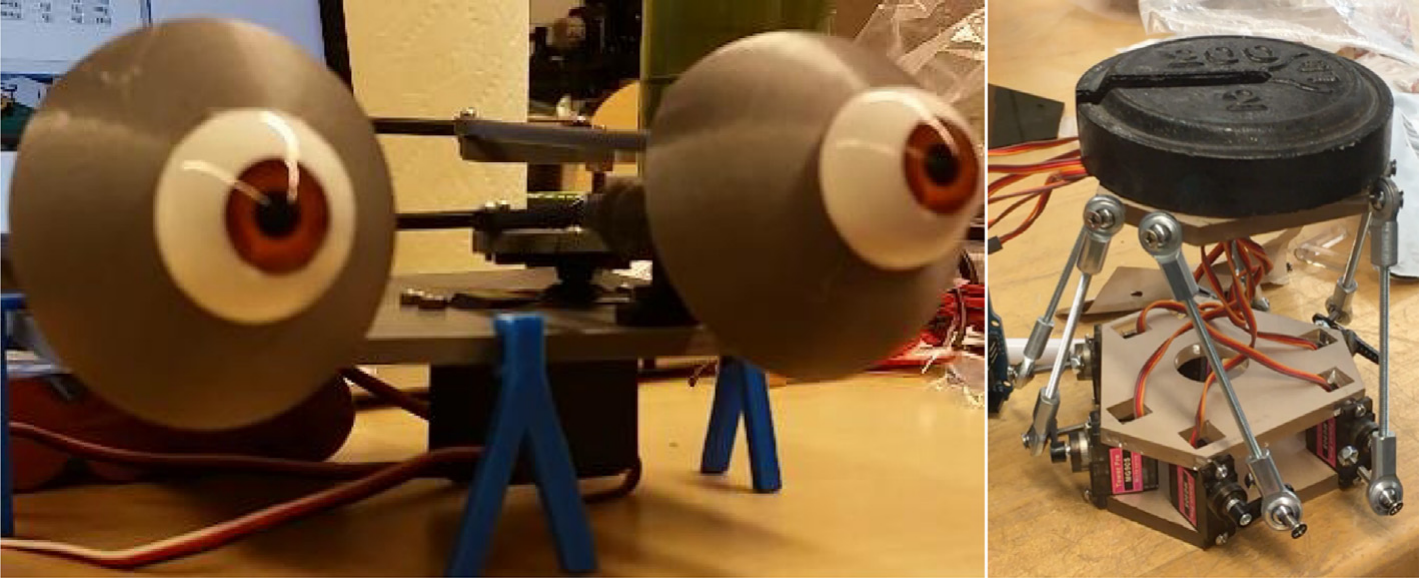
(left) Early prototype of eye mechanism based on an open-source design by Polymaker, (right) early prototype of neck mechanism supporting a test weight.

Another informative prototype was an early prototype of the eye mechanism, as shown in Fig. 18. This design, which was largely based on an open-source design by Polymaker [15], uses two servo motors and is capable of simultaneously rotating both eyeballs vertically and horizontally, with the vertical rotation being independent of the horizontal rotation. However, the eyeballs in this prototype do not rotate about their centroid, and thus translate horizontally or vertically when rotating. When placed inside the skull, this translation would cause the eyeballs to collide with the 3D printed skull (face). Thus, the final eye mechanism was designed to eliminate translation by rotating the eyeballs about their centroids.

Finally, an early prototype of the neck mechanism, also shown in Fig. 18, revealed that a standard Stewart platform would be unable to support the weight of the skull and its internal components. The servo motors in this prototype had reached their stall torque just from supporting the weight on the top plate. While a standard Stewart platform with servo motors is suited for precise control at light loads, Eva’s neck mechanism needs to support a relatively large load. To achieve this performance, three concentric springs were placed between the middle and top plates to partially support the load of the head. While slightly reducing the mechanism’s precision and range of motion, the springs greatly reduce the load on the servo motors and allow the top platform to move as expected even when supporting a weight three times larger than the weight of the full Eva assembly.

Another major design decision informed by these early prototypes was the decision of how closely Eva should resemble a real human. According to the uncanny valley theory proposed by Masahiro Mori, while humans have an affinity, or fondness, for toy robots and other humans, realistic humanoids that imperfectly resemble humans elicit a feeling of revulsion and eeriness from human observers [16]. According to Mori, as a humanoid becomes more realistic, the emotional response it elicits from a human observer generally becomes more positive. However, there is a distinct valley in which increasing the realism of the humanoid actually makes it more unsettling. Mori hypothesized that this phenomenon could be a result of the human instinct to avoid diseased individuals. When a humanoid is highly realistic but possesses some obvious aesthetic flaws, it might resemble a diseased individual, thus causing a feeling of unease in human observers.

The exact nature and cause of the uncanny valley phenomenon is still under debate but the phenomenon has been observed and recreated in a variety of settings since Mori’s original hypothesis in 1970 [17]. Thus, avoiding the uncanny valley was a major requirement for Eva’s design. To avoid the uncanny valley, Eva would either have to appear highly realistic with only minor flaws (past the uncanny valley) or intentionally simplistic and mechanical (before the uncanny valley). Creating a highly realistic humanoid was not feasible given the time and resource restrictions of this project. Thus, we designed Eva to lie before the uncanny valley so that it appears human but can still be clearly recognized as a robot through its visible mechanical components. This effect was achieved in several ways. Based on the prototype with the latex mask, which had a somewhat unsettling appearance due to its realistic skin tone, we decided to dye the silicone mask blue to convey that Eva’s skin is artificial. We also highlighted Eva’s mechanical nature by exposing the entirety of the neck mechanism, the rear of the 3D printed skull, and the wires that connect to the Raspberry Pi during operation. Finally, the absence of a torso or body further indicates that Eva is clearly a robot rather than a real human. However, due to the subjective nature of the uncanny valley phenomenon, some human observers may still find Eva’s appearance unsettling.

Another way in which we designed Eva to be more pleasant to interact with was by giving it a feminine name and a somewhat feminine facial structure. According to a 2009 study that examined the interactions between human participants and humanoids, female humanoids are generally perceived to be slightly more credible, trustworthy, and engaging than male humanoids [18]. Thus, as Eva was designed to engage and interact with humans, we found it appropriate to designate Eva as female rather than male. With that said, Eva’s gender was a relatively minor design consideration. We decided to focus our efforts on improving Eva’s functionality rather than giving it an overtly feminine appearance, relying on its feminine name to communicate its gender.

## 6 Operation instructions

### 6.1 Operating procedure

The operation of Eva is fairly simple and intuitive. The following procedure outlines a typical work session.

1. Pick up Eva by the acrylic base platform using both hands and place it on a level surface for the work session.
2. Connect an external monitor to the Raspberry Pi using an HDMI cable.
3. Connect an external keyboard and mouse to the Raspberry Pi using their corresponding USB cables.
4. Power on the Raspberry Pi by connecting its 2.5A power supply via the Micro USB port.
5. Turn on the external monitor and use the keyboard and mouse to navigate to the Desktop on the Raspberry Pi.
6. Connect the Raspberry Pi to a Wi-Fi network if an internet connection is necessary.
7. Open an IDE for programming Eva in your preferred language. Note that Python is the recommended language for working with Eva as Eva’s functionality was tested exclusively in Python.
8. Write your code.
9. Before running code that makes use of the servo motors, connect the 5 V 10A power supply to the Raspberry Pi using its 2.1 mm DC plug.
10. Run your code and make sure to disconnect the 10A power supply when you no longer need to use the servo motors.
11. To end your current work session, stop any program that is currently executing, turn off the Raspberry Pi, and disconnect the power supplies, monitor, keyboard, and mouse.
12. Pick up Eva by the acrylic base platform using both hands and place it in a safe location for storage.

### 6.2 Potential operating hazards

The main safety hazards that can arise when operating Eva are overheating and electrical failure. The cooling fan attached to the top of the skull is designed to keep the internal electronics at a safe temperature, but overheating may occur during excessively long work sessions where the servo motors are in constant use. Thus, researchers should try to limit their sessions to a few hours at a time and avoid consistent, heavy use of the servo motors. The risk of overheating is also exacerbated when operating Eva in an environment with a high ambient temperature. Eva should ideally be operated exclusively in a cool, air-conditioned room away from direct sunlight.

The other main safety hazard, electrical failure, can occur when using an incorrect power supply or using untested electronic components and cables. The only power supplies that have been approved for Eva are the standard 2.5A power supply used to power the Raspberry Pi and the 5 V 10A power supply specified in the Bill of Materials that is used to power the servos and cooling fan. The 10A power supply is sufficient for powering all of Eva’s servos and can support a few additional servos and sensors such as cameras and microphones. Using more powerful power supplies could damage the Raspberry Pi and any connected electronics. Furthermore, make sure that all cables and peripheral electronics have been tested by a regulatory body such as UL.

## 7 Validation and characterization

### 7.1 Demonstration of functionality

Figs. 19a, 19b and 19c below demonstrate Eva’s mechanical capabilities. In addition to the facial expressions and movements shown in the figures, Eva can also emit text-to-speech audio through its onboard speaker. For a complete demonstration of Eva’s capabilities, refer to the video titled “Eva Demo.mp4” as referenced in Section 3. This video is able to more clearly illustrate Eva’s capabilities than the following still photographs.

**Fig. 19a:**
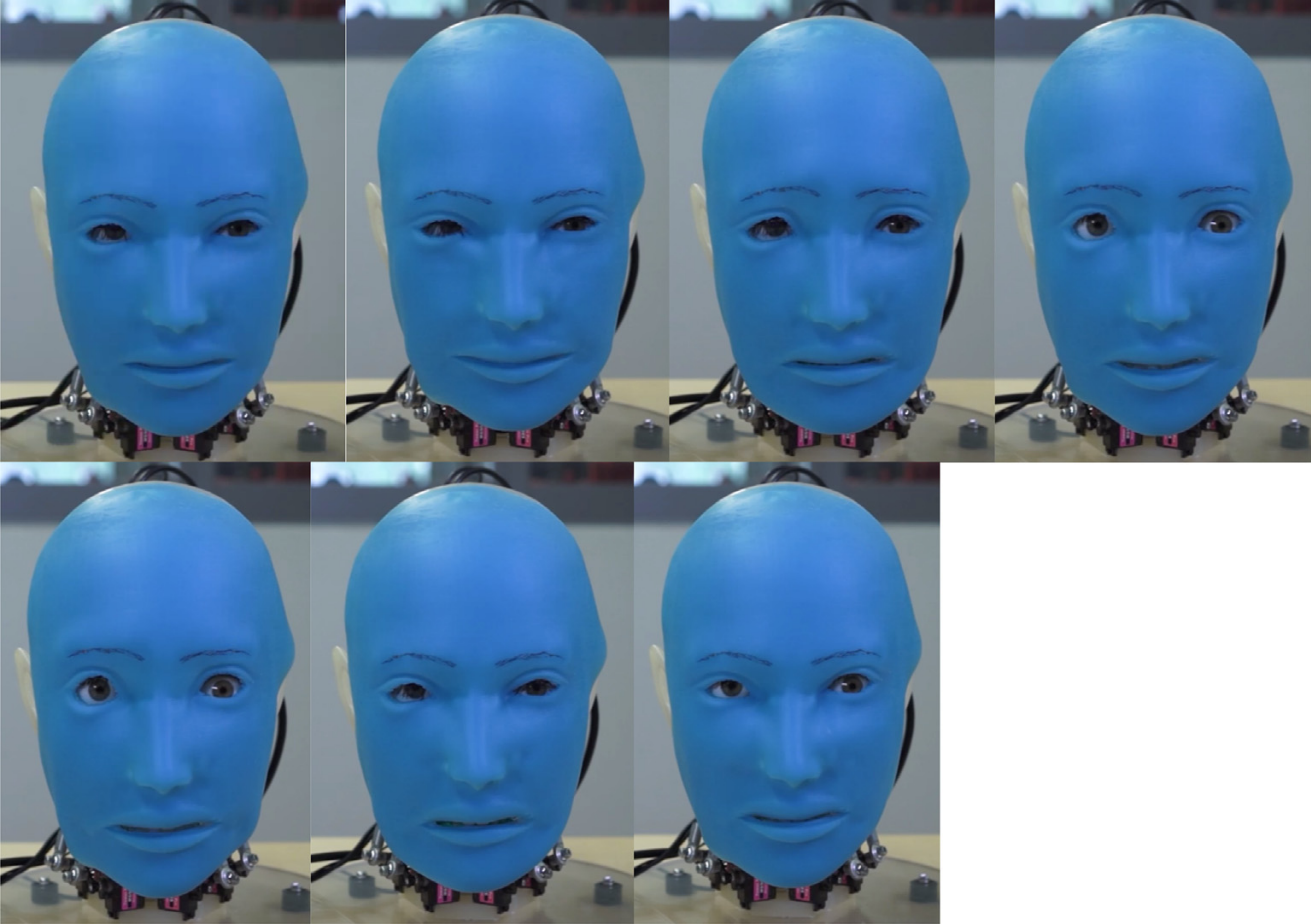
Facial expressions from left to right: (1) neutral, (2) happy, (3) sad, (4) surprised, (5) afraid, (6) disgusted, (7) angry.

**Fig. 19b:**
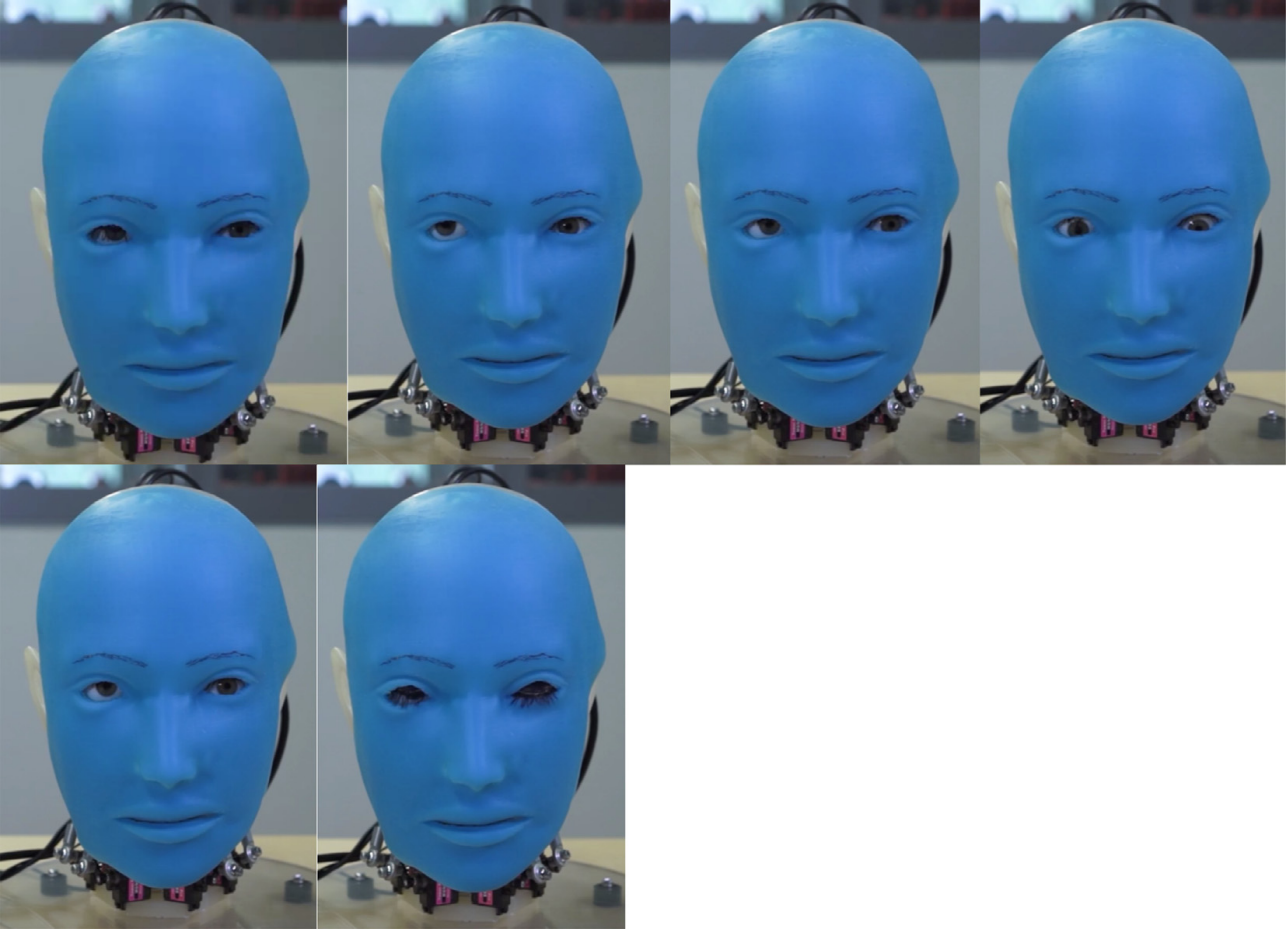
Eye movements from left to right: (1) neutral, (2) look right, (3) look left, (4) look down, (5) look up, (6) close eyelids.

**Fig. 19c:**
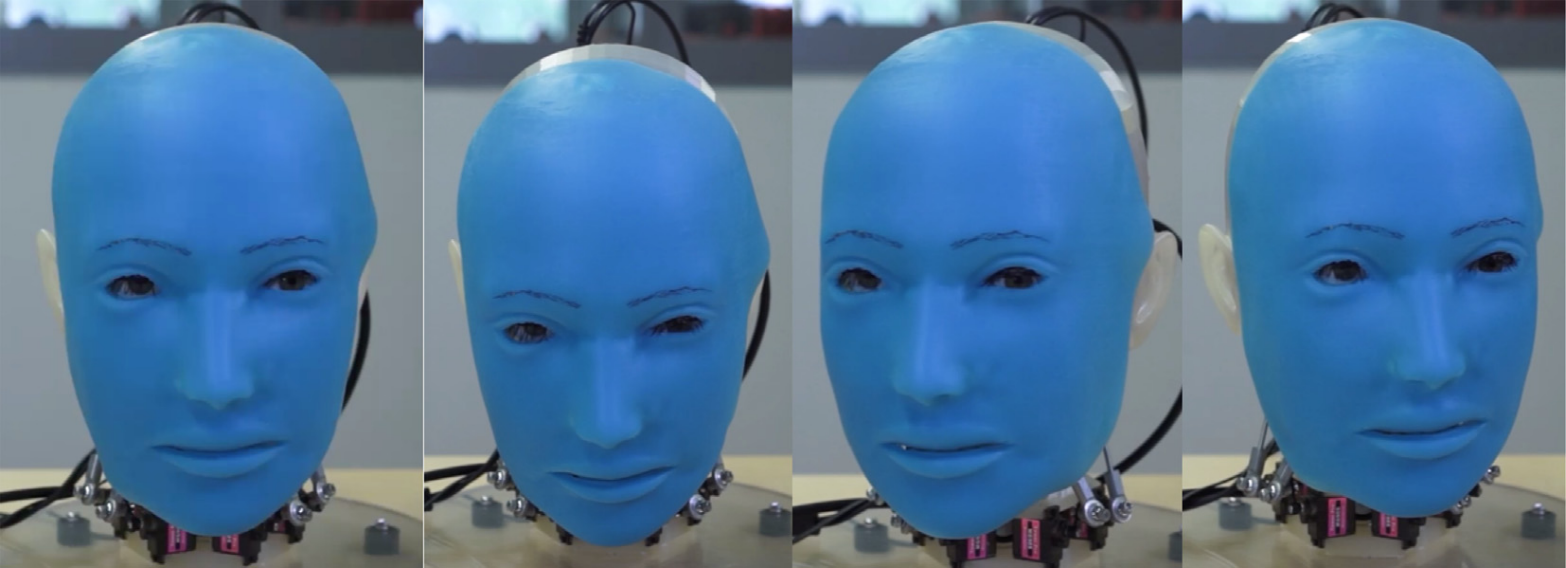
Neck movements from left to right: (1) neutral, (2) tilt downwards, (3) turn right, (4) turn left.

### 7.2 Limitations and future work

Eva’s facial expressions and head movements are functional but additional work must be completed before Eva can serve as a platform for substantive AI research. In particular, as Eva currently has no sensors to take in data from its environment, it is currently incapable of responding or reacting to humans. To overcome this limitation, researchers could incorporate a camera and a microphone so that Eva can record video and audio when interacting with humans. This video and audio data can then be used in machine learning algorithms so that Eva can learn to recognize the emotions of its human conversational partners and understand how its own facial expressions, movements, and speech affect the emotional state of humans. Once equipped with sensors, Eva should be effective at interacting with humans in a one-on-one setting, where the human sits or stands in front of Eva and communicates with the robot through verbal speech, facial expressions, and body language.

Additionally, several improvements can be made to Eva’s current functionality. In particular, the degree to which the jaw mechanism is able to open the mouth is less than initially expected. This is a result of the large force required to stretch the mask enough to visibly open the mouth. In future iterations of Eva, this problem can be addressed by using more powerful servo motors for the jaw mechanism, using a thinner mask, or adding weight to the jaw so that the mask is already loaded before the servo motors apply force to rotate the jaw. Eva’s functionality could be further improved by reducing the noise emitted by its servo motors. Currently, Eva’s motors produce a significant amount of noise that may be distracting to a human observer. While this distraction can be mitigated by increasing the volume of Eva’s onboard speaker, researchers may want to decrease the noise by replacing the current MG90S servos with servos that operate more quietly. Finally, to further reduce the risk of overheating and increase the time Eva can be continuously operated, researchers may want to consider improving Eva’s cooling mechanism. Specifically, the cooling mechanism could be improved by adding additional fans, incorporating heat sinks, or adding holes to the 3D printed skull to increase air circulation.

Eva has several other limitations that can be addressed relatively easily with software modifications rather than hardware modifications. To make the surprised and afraid expressions more distinct, researchers can adjust the degree to which the AUs in these two expressions are actuated by the corresponding servo motors. For example, AU 5, which corresponds to the opening of the eyelid, can be more exaggerated for the afraid expression than the surprised expression. Refer to Section 5 for more information on AUs and their correspondence to facial expressions. Additionally, the movement of the neck can be made smoother by modifying the control system for the Stewart platform of the neck mechanism. Finally, the coordination between the text-to-speech audio and the opening and closing of the mouth can be improved to enhance the illusion of speech.

### 7.3 Performance metrics

Eva’s performance was largely based on a qualitative evaluation of its ability to mimic human facial expressions and movements, but a few key performance metrics were recorded to aid in the design process and in the evaluation of its performance. These metrics are listed below.

• The neck mechanism can safely support a load of 4.54 kg. As the current weight of the components the neck must support in the full assembly is approximately 1.37 kg, additional components can still be added inside the skull without negatively affecting the performance of the neck mechanism.
• The maximum current draw of the servo motors and cooling fan during normal operation is 7.65 A to 9.17 A, with 7.65 A indicating a reasonable maximum estimate and 9.17 A indicating an unlikely, absolute maximum. Both the servo motors and the cooling fan are powered through the Adafruit servo hats, which are rated to accept a maximum current of 10 A at 5 V. The 5 V 10 A power supply listed in the Bill of Materials is thus able to adequately power the servo motors and cooling fan and can support a few additional components such as a camera and microphone, depending on their maximum current draw.
• The maximum tested time for the continuous operation of Eva is 3 h. This test was conducted in a cool, air-conditioned room with Eva cycling through all of its facial expressions, movements, and speech capabilities.
• The horizontal and vertical range of motion of the eyeballs is approximately 45 degrees each.
• The range of motion of the eyelids is approximately 70 degrees each.
• The range of motion of the jaw is approximately 15 degrees. This is considerably less than initially expected, as explained in Section 7.2.
• The vertical range of motion of the neck is approximately 30 degrees and its horizontal range of motion is approximately 45 degrees.

## Declaration of Competing Interest

The authors declare that they have no known competing financial interests or personal relationships that could have appeared to influence the work reported in this paper.
